# PhoU2 but Not PhoU1 as an Important Regulator of Biofilm Formation and Tolerance to Multiple Stresses by Participating in Various Fundamental Metabolic Processes in Staphylococcus epidermidis

**DOI:** 10.1128/JB.00219-17

**Published:** 2017-11-14

**Authors:** Xiaofei Wang, Haiyan Han, Zhihui Lv, Zhiwei Lin, Yongpeng Shang, Tao Xu, Yang Wu, Ying Zhang, Di Qu

**Affiliations:** a Department of Medical Microbiology and Parasitology, Key Laboratory of Medical Molecular Virology of MOE and MOH, School of Basic Medical Sciences, Fudan University, Shanghai, China; b Key Laboratory of Medical Molecular Virology, Huashan Hospital, Shanghai Medical College of Fudan University, Shanghai, China; c Department of Molecular Microbiology and Immunology, Bloomberg School of Public Health, Johns Hopkins University, Baltimore, Maryland, USA; d Department of Laboratory Medicine in the Second Affiliated Hospital of Zhengzhou University, Zhengzhou, China; Michigan State University

**Keywords:** biofilm, Staphylococcus epidermidis, tolerance

## Abstract

PhoU, a conserved protein that has been proposed to coordinate phosphate import, is a negative regulator of drug tolerance in most bacteria. In Staphylococcus epidermidis, the role of PhoU in biofilm formation and drug tolerance has not yet been investigated. Two PhoU homologs in the genome of S. epidermidis have been identified by the presence of the conserved motif E(D)XXXD of PhoU. We separately constructed Δ*phoU1* and Δ*phoU2* mutants of S. epidermidis strain 1457. The Δ*phoU2* mutant displayed growth retardation, a weakened biofilm formation capacity, a higher sensitivity to H_2_O_2_, and reduced tolerance to multiple antibiotics. However, deletion of *phoU1* had no effect on those. We compared the transcriptome profiles of the Δ*phoU2* and Δ*phoU1* mutants with that of the parent strain. In the Δ*phoU2* mutant, expression of genes related to inorganic phosphate uptake was significantly upregulated (*pst* operon) and the levels of intracellular inorganic polyphosphate (polyP) were increased. In the Δ*phoU2* mutant, expression of enzymes in the pentose phosphate pathway (PPP) was downregulated and less NADP (NADPH) was detected, consistent with the high sensitivity to H_2_O_2_ and the growth retardation of the Δ*phoU2* mutant. The upregulated expression of ATP synthase was consistent with the high intracellular ATP content in the Δ*phoU2* mutant, which may have been related to the lower drug tolerance of the Δ*phoU2* mutant. This study demonstrates that PhoU2, but not PhoU1, in S. epidermidis regulates bacterial growth, biofilm formation, oxidative stress, and drug tolerance in association with alterations to inorganic phosphate metabolism, the pentose phosphate pathway, galactose metabolism, the tricarboxylic acid (TCA) or citric cycle, glycolysis and gluconeogenesis, and respiratory reactions.

**IMPORTANCE** PhoU is widely conserved throughout the bacterial kingdom and plays an important role in response to stress and metabolic maintenance. In our study, two PhoU homologs were found in S. epidermidis. The function of *phoU2*, but not *phoU1*, in S. epidermidis is related to growth, drug tolerance, the oxidative stress response, polyP levels, and ATP accumulation. In addition, *phoU2* regulates biofilm formation. Hence, *phoU2* is a regulator of both drug tolerance and biofilm formation, which are two bacterial properties that present major challenges to the clinical treatment of infections. Analysis of differential gene expression revealed that *phoU2* is involved in fundamental metabolic processes, such as the PPP pathway. These findings indicate that *phoU2* is a crucial regulator in S. epidermidis.

## INTRODUCTION

Staphylococcus epidermidis is a common opportunistic pathogen that is present on human skin and mucosal surfaces. The pathogenicity of S. epidermidis is mainly due to biofilm formation on foreign devices, such as catheters, heart valves, and prostheses (1, 2). The bacteria within the biofilm are protected against killing by antibiotics and the host immune system, contributing to the increasing emergence of resistance to antimicrobial drugs and to the establishment of persistent infections (3, 4). PhoU, the phosphate transport system regulatory protein, is now known to be a negative regulator of drug tolerance in Escherichia coli, Mycobacterium tuberculosis, and Pseudomonas aeruginosa (5–8), whereas the possible role of PhoU in biofilm formation and drug tolerance in S. epidermidis has not been investigated.

On the basis of a crystal structure analysis of PhoU in Thermotoga maritima (9), multinuclear iron clusters [E(D)XXXD] have been identified to be the conserved motif of the protein. According to the conserved motif, one homolog of *phoU* is found in E. coli, P. aeruginosa, and Streptococcus, while two *phoU* homologs are found in T. maritima, M. tuberculosis, Mycobacterium marinum, and Staphylococcus aureus. In E. coli and P. aeruginosa, *phoU* is located in the *pst* operon, which contains four other genes: *pstS*, *pstA*, *pstC*, and *pstB*. PstS is a periplasmic inorganic phosphate (P_i_)-binding protein that captures and transfers P_i_ to the channel formed by the integral proteins PstC and PstA in the cytoplasmic membrane. PstB is an ATPase that provides the energy for transport (10). The last gene in the operon, *phoU*, encodes a protein that does not have a defined function. In 2007, it was reported that *phoU* in E. coli plays an important role in the development of multidrug-tolerant bacteria (5). In P. aeruginosa, *phoU* is a negative regulator of intracellular ppGpp and polyphosphate (polyP), but it does not regulate biofilm formation (8). In M. tuberculosis and M. marinum, there are two *phoU* homologs in the genome, *phoY1* and *phoY2*. However, neither *phoY1* nor *phoY2* is located in the *pst* operon. In the two mycobacterial species, *phoY2* was shown to be a functional homolog of *phoU*, regulating the generation of multidrug-tolerant bacteria and maintaining metabolic homeostasis and adaptation to stress conditions (6, 7). In the S. aureus (NCTC8325) genome, there are two *phoU* homologs (*SAOUHSC_01384* and *SAOUHSC_00669*). In S. aureus, *SAOUHSC_01384* resides in the *pst* operon as *phoU*, while *SAOUHSC_00669* (*pitR*) also contains the conserved motif of *phoU* and is located upstream of *pitA* (a phosphate uptake gene). A point mutation in *pitA* in S. aureus resulted in bacteria that were sensitized to daptomycin, and SAOUHSC_00669 was found to be required for daptomycin sensitivity (11, 12). In the present study, protein motif analysis of S. epidermidis ATCC 35984 revealed two genes that were homologous to *phoU* (*serp0956* and *serp0316*). *serp0956*, found in the *pst* operon, was named *phoU1*, while *serp0316*, located upstream of a hypothetical protein with a high degree of homology to *pitR* of S. aureus, was named *phoU2*.

In staphylococci, biofilm formation and drug tolerance are regulated by multiple regulatory factors (13, 14). In this study, we focused on investigating the roles of PhoU homologs in the biofilm formation and drug tolerance of S. epidermidis. In S. epidermidis strain 1457 (SE1457), either *phoU1* or *phoU2* was deleted by allelic replacement to create the Δ*phoU1* and Δ*phoU2* mutants, respectively. The effects of these deletions on bacterial growth, biofilm formation, drug tolerance, and oxidative stress were investigated. Comparison of the transcriptomes of the Δ*phoU2* mutant and the parent strain revealed the differentially expressed genes (DEGs) involved in the pentose phosphate (PPP) pathway, galactose metabolism, the trichloroacetic acid (TCA) cycle, glycolysis and gluconeogenesis, and respiratory chain reactions. The involvement of some of the DEGs in metabolic processes, such as polyP accumulation, ATP accumulation, and the pentose phosphate pathway, was validated. We conclude that *phoU2* probably regulates bacterial growth, biofilm formation, oxidative stress responses, and drug tolerance in S. epidermidis. In contrast, *phoU1* has no obvious effect on biological activity in S. epidermidis.

## RESULTS

### The two *phoU* loci identified in the genome of S. epidermidis.

**FIG 1:**
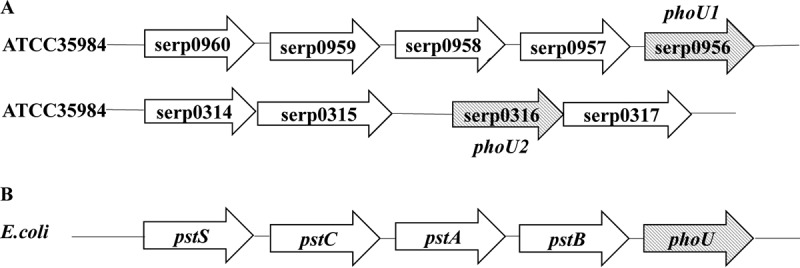
Two genes homologous to *phoU*, *phoU1* and *phoU2*, were found in S. epidermidis RP62A by motif analysis according to the PhoU conserved motifs [E(D)XXXD] of Thermotoga maritima. (A) Genetic locations of *phoU1* and *phoU2* in S. epidermidis RP62A. (B) Genetic organization of the *pst* operon and *phoU* in E. coli.

In the genome of the S. epidermidis ATCC 35984 strain (GenBank accession number CP000029), two PhoU gene homologs, *serp0956* and *serp0316*, were identified by bioinformatics analysis (https://www.ncbi.nlm.nih.gov/genome/?term=RP62a) on the basis of the conserved motif E(D)XXXD of Thermotoga maritima. *serp0956* is located in the *pst* operon, similarly to the *phoU* gene in E. coli, which is denoted as encoding a phosphate transport system regulatory protein and designated *phoU1* in this study. *serp0316*, denoted *phoU2*, encodes a hypothetical protein, is located far from the *pst* operon, and was cotranscribed with the adjacent gene, *serp0317* (Fig. 1). The two PhoU homologs in S. epidermidis shared 12.5% identity and 30.1% consensus sequences.

### Construction of *phoU1* and *phoU2* deletion strains.

To identify the function of *phoU1* or *phoU2* in S. epidermidis, mutants of the SE1457 strain with a deletion of *phoU1* or *phoU2* were constructed using the temperature-sensitive plasmid pKOR1. The deletion mutants were verified by PCR, reverse transcription (RT)-quantitative PCT (qPCR), and direct sequencing and are referred to as the Δ*phoU1* and Δ*phoU2* mutants, respectively. The complemented Δ*phoU2* strain was constructed using shuttle vector pCN51 and named SE1457 Δ*phoU2*/pCN51::*phoU2*. The Δ*phoU*2 strain containing the empty vector pCN51 was designated SE1457 Δ*phoU2*/pCN51.

### Growth curves of the Δ*phoU1* and Δ*phoU2* mutants.

To evaluate the effect of *phoU1* or *phoU2* knockout on the growth of S. epidermidis, growth curves of the Δ*phoU1* and Δ*phoU2* mutants and the parent strain (SE1457) were generated under oxic and microaerobic conditions.

**FIG 2:**
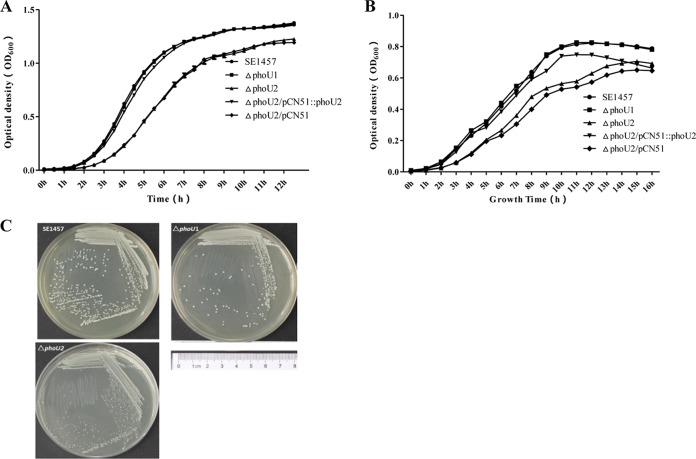
Effect of *phoU2* deletion on the growth of S. epidermidis. Overnight cultures of the Δ*phoU1*, Δ*phoU2*, and SE1457 strains were diluted 1:200 into 10 ml TSB in a conical flask in a volume of 100 ml and incubated with shaking at 220 rpm. Bacterial growth was monitored by measuring the OD_600_ for 12 h. Data (means ± SDs) are from three independent experiments. (A) Growth curves of the Δ*phoU1* and Δ*phoU2* mutants under oxic conditions. (B) Growth curves of Δ*phoU1* and Δ*phoU2* mutants under microaerobic conditions. (C) Representative images showing the colony morphology of the Δ*phoU1* and Δ*phoU2* mutants and the SE1457 parent strain grown on TSB agar plates at 37°C for 24 h under oxic conditions. The results represent those from one of three independent experiments.

Under oxic conditions, in comparison to the parent strain, the Δ*phoU2* mutant displayed a marked reduction in growth (Fig. 2A). However, the Δ*phoU1* mutant displayed growth curves and colony sizes similar to those of the parent strain when the strains were incubated under the same conditions. By culture in liquid tryptic soya broth (TSB) medium at 37°C for 6 h, the optical density at 600 nm (OD_600_) of the Δ*phoU2* mutant reached 1.26 ± 0.343, and that of the parent strain was 2.41 ± 0.078. On solid culture medium (TSB agar), the colony size of the Δ*phoU2* mutant was much smaller than that of the parent strain (Fig. 2C). The expression of *phoU2* by the pCN51 vector restored the growth of the Δ*phoU2* mutant to the level of the parent strain.

Under microaerobic conditions, the growth rates of the Δ*phoU1* and Δ*phoU2* mutants and SE1457 in liquid medium were measured by recording the OD_600_ of the cultures. After 6 h of incubation, the OD_600_ of the Δ*phoU2* mutant was 0.265 ± 0.04, which was much less that than that of SE1457 (0.418 ± 0.03) (Fig. 2B). However, no differences in the growth curve could be detected in the Δ*phoU1* mutant when the strains were incubated under the same conditions.

### Morphology and autolysis of the Δ*phoU1* and Δ*phoU2* mutants.

**FIG 3:**
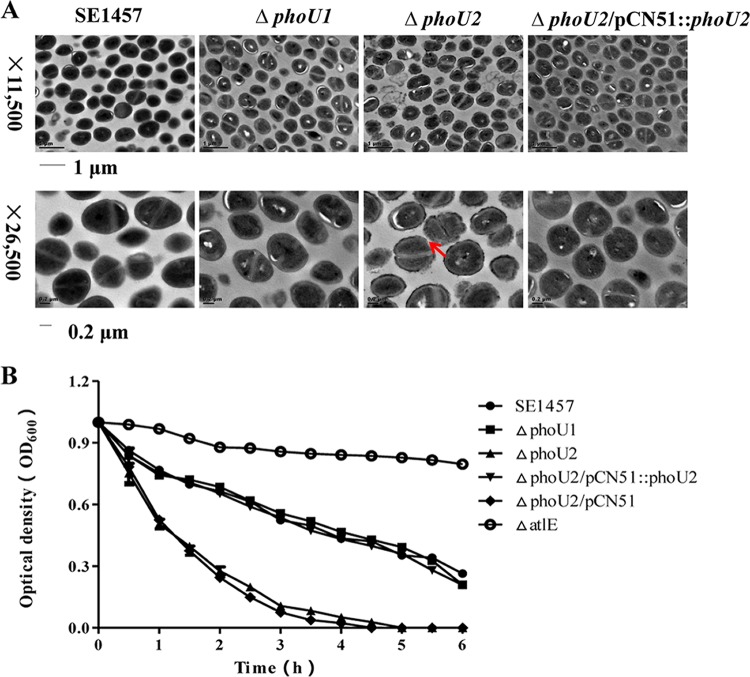
(A) Bacterial morphology of the Δ*phoU1* and Δ*phoU2* mutants observed by TEM. The ultrastructure of the log-phase bacteria was observed by transmission electron microscopy (Philips Tecnai-12 Biotwin). Arrow, disruption of the cell wall in the Δ*phoU2* mutant. (B) Autolysis of the Δ*phoU1* and Δ*phoU2* mutants induced by Triton X-100. Overnight cultures were suspended in Triton X-100 autolysis buffer (50 mM glycine, pH 8.0, containing 0.01% Triton X-100) to an initial OD_600_ of approximately 1.0, and the rates of autolysis were monitored on the basis of the decrease in the OD_600_ value over time.

The morphologies of the Δ*phoU1* and Δ*phoU2* mutants and the parent strain were observed using transmission electron microscopy (TEM). A rough cell wall and thin cytoplasm were observed in the Δ*phoU2* mutant (magnification, ×26,500), while the morphology of the Δ*phoU1* mutant was similar to that of the parent strain (Fig. 3A).

The autolysis capacity of the Δ*phoU1* and Δ*phoU2* mutants and the parent strain was then assessed by Triton X-100 induction. Mid-exponential-phase cultures (6 h) of the Δ*phoU1* and Δ*phoU2* mutants and SE1457 were adjusted to an OD_600_ of 1.0 and incubated with 0.05% Triton X-100 for 5 h, and the autolysis rate was measured by determination of the OD_600_ using a spectrophotometer. After Triton X-100 induction, the autolysis rate of the Δ*phoU2* mutant reached 88% after treatment for 3 h, which was significantly higher than that of SE1457 (46%). The autolysis rate of Δ*phoU1* (43%) was similar to that of the parent strain (Fig. 3B).

### Biofilm formation of the Δ*phoU1* and Δ*phoU2* mutants under static or hydrodynamic conditions.

**FIG 4:**
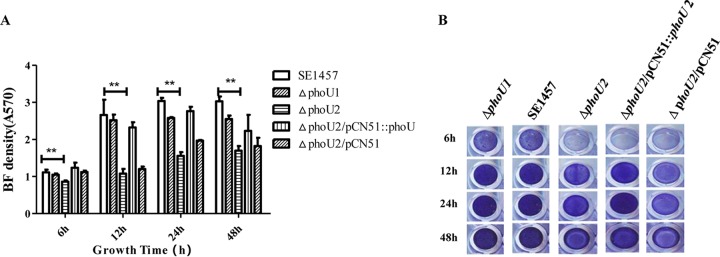
Biofilm (BF) formation by the Δ*phoU1* and Δ*phoU2* mutants on microtiter plates. Overnight cultures of the S. epidermidis strains were diluted 1:200 with fresh TSB, added to 96-well polystyrene plates in triplicate, and cultured under static conditions for 6 h, 12 h, 24 h, and 48 h. After the biofilms were washed, they were stained with crystal violet. The OD_570_s of the plates were analyzed. The experiments were repeated three times, and the data represent means ± SDs. **, *P* < 0.01.

Under static conditions, the polystyrene microtiter plate assay and the confocal laser scanning microscopy (CLSM) observation assay were performed to evaluate the role of *phoU1* or *phoU2* in biofilm formation. Bacterial biofilm formation was monitored at 6, 12, 24, and 48 h on microtiter plates stained with crystal violet, and the OD_570_ was read. The mature biofilm of the Δ*phoU2* mutant (OD_570_, 1.56 ± 0.10) was significantly decreased compared with that of the parent strain (OD_570_, 3.04 ± 0.08) after incubation for 24 h, and the Δ*phoU1* deletion (OD_570_, 2.58 ± 0.02) had little effect on biofilm formation (Fig. 4). The complemented Δ*phoU2/*pCN51::*phoU2* strain (OD_570_, 2.77 ± 0.12) showed a restored biofilm formation ability.

**FIG 5:**
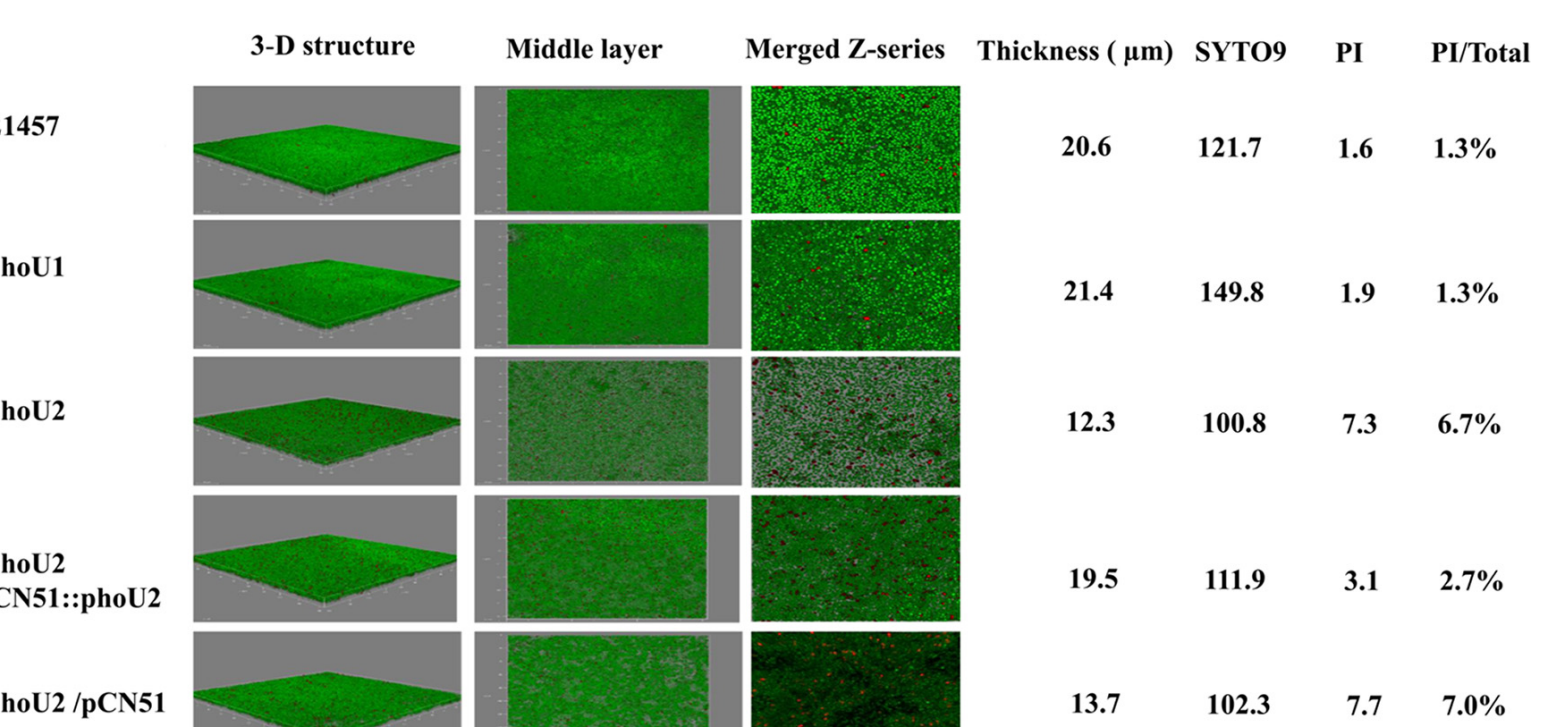
Biofilms of the Δ*phoU1* and Δ*phoU2* mutants observed by CLSM. Twenty-four-hour-old biofilms of SE1457 and the Δ*phoU1* and Δ*phoU2* mutants were grown on a cover glass in a cell culture dish and observed by CLSM. Three-dimensional (3-D) structural images (zoom 1, ×63 magnification) were reconstructed, and the thickness of the biofilms was measured using Imaris software. Viable and dead cells were stained green (SYTO9) and red (PI), respectively. The amount of fluorescence in the middle layer of the biofilm was determined using ImageJ software (zoom 3, ×63 magnification). The PI/total florescence value indicates the proportion of dead cells within the biofilm. The images and values are representative of those from one of three independent experiments.

Furthermore, the biofilm formation ability of the Δ*phoU2* mutant *in vitro* was examined by confocal laser scanning microscopy. After incubation at 37°C for 24 h, SE1457 formed a compact, thick biofilm on a glass coverslip in a cell culture dish. In contrast, the biofilm of the Δ*phoU2* mutant was much thinner than that of the parent strain. Additionally, the dead cell ratio in the biofilm of the Δ*phoU2* mutant (6.7%) was 5-fold higher than that in the biofilm of the parent strain (1.3%) (Fig. 5).

**FIG 6:**
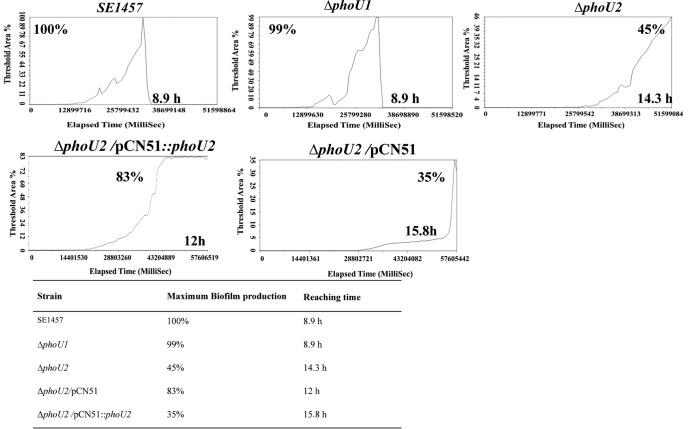
Biofilm formation by the Δ*phoU1* and Δ*phoU2* mutants under hydrodynamic conditions. Overnight cultures of the S. epidermidis strains were diluted 1:200 with fresh TSB and added to BioFlux 48-well plates. The bacteria were then cultured under hydrodynamic conditions with a shear setting of 0.15 dyne/cm^2^. A BioFlux 1000 system (Fluxion Biosciences) with a Leica microscope and temperature-controlled housing was used for all imaging experiments. Automated microscopy and image processing were performed with BioFlux Montage software. Images were automatically acquired every 10 min at multiple stage positions with bright-field illumination; images were also acquired in the red channel using a 200-ms exposure time. The background-corrected average pixel intensity per image was used to quantify the biofilm formation by the different strains. The curve was generated on the basis of the images. A synthesis of the images is shown in Movies S1 to S5 in the supplemental material. Each figure represents the results of one of three independent experiments.

Under hydrodynamic conditions, the biofilm formation of the Δ*phoU2* mutant strain was analyzed using a BioFlux 1000 device with a flow speed of 0.15 dyne/cm^2^. The maximum size of the biofilm of the Δ*phoU2* mutant observed in the channel was 2-fold smaller than that of the biofilm of the parent strain and the complemented strain, and the time required to form the maximum biofilm was 5.4 h longer for the Δ*phoU2* mutant strain than for the parent strain (Fig. 6). The biofilm formation of the Δ*phoU1* mutant in the hydrodynamic state appeared to be similar to that of the parent strain. The complemented Δ*phoU2/*pCN51::*phoU2* strain could partially restore the biofilm formation capacity under hydrodynamic conditions.

**FIG 7:**
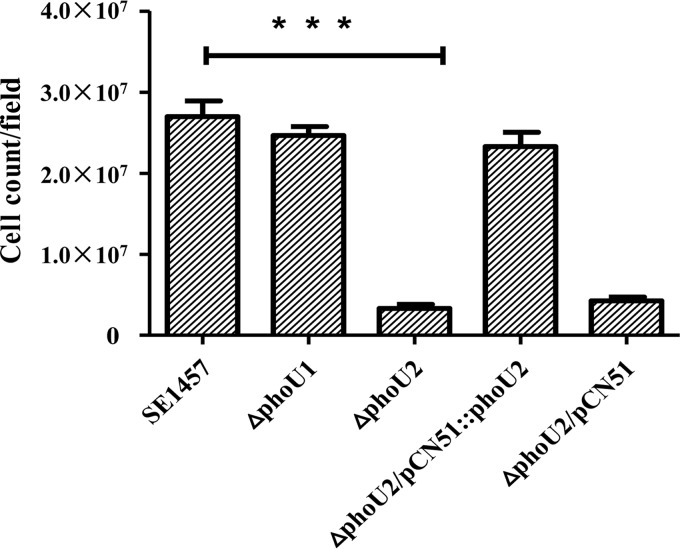
Initial attachment of the Δ*phoU1* and Δ*phoU2* mutants on polystyrene plates. Log-phase bacterial cultures in TSB were adjusted to an OD_600_ of 0.1 with PBS, and 5-ml aliquots were added to a 6-well polystyrene plate. After incubation at 37°C for 30 min, each well was washed three times with PBS, and the adhered cells were observed and photographed. The amounts of attached bacterial cells of the Δ*phoU1* and Δ*phoU2* mutants and SE1457 are indicated. The results (means ± SDs) are from three independent experiments. ***, *P* < 0.001.

The initial attachment phase is characterized as the first phase of the process of Staphylococcus epidermidis biofilm formation. The initial attachment capacity of the Δ*phoU2* mutant was determined, and the attached cells were counted using ImageJ software. There were fewer attached cells for the Δ*phoU2* mutant (3.32 × 10^6^) than for the parent strain (2.70 × 10^7^) and the complemented strain Δ*phoU2/*pCN51::*phoU2* (2.33 × 10^7^). The number of attached cells of the Δ*phoU1* mutant (2.47 × 10^7^) was similar to the number of attached cells of the parent strain (Fig. 7).

**FIG 8:**
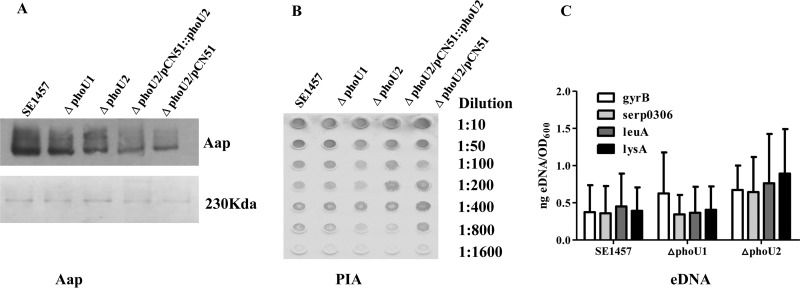
Effects of the Δ*phoU1* and Δ*phoU2* mutants on extracellular matrix biosynthesis by S. epidermidis. (A) Aap expression in the Δ*phoU1* and Δ*phoU2* mutants. Twenty-four-hour-old biofilms and 12-h-old planktonic bacteria were collected after they were washed with PBS. Lysostaphin-treated samples with identical OD_600_s were centrifuged at 20,000 × *g* for 30 min at 4°C. The supernatant was separated by 7% SDS-PAGE, and the gel pieces containing Aaps were used for Western blotting (top).The remaining gel pieces were stained with Coomassie blue as an endogenous reference (bottom). MAb_18B6_ (10 ng/ml) was used as the primary antibody. Immunoreactivity was detected using an ECL Western blotting system after incubation with HRP-conjugated secondary antibody. (B) PIA biosynthesis was semiquantified using a dot blot assay with WGA. Twenty-four-hour-old biofilms were scraped off and suspended in EDTA. Serial dilutions of the PIA assay extracts were spotted onto nitrocellulose membranes, subsequently incubated with WGA conjugated to HRP, and visualized by chromogenic detection. (C) eDNA quantified by qPCR of four chromosomal loci (*gyrB*, *serp0306*, *leuA*, and *lysA*). The OD_600_s of unwashed 24-h-old biofilms were measured for normalization to the biofilm biomass, and then the biofilms were used for eDNA isolation by phenol-chloroform-isoamyl alcohol extraction and ethanol precipitation. The results are presented as the amount of eDNA per biofilm biomass (means ± SDs) from three independent experiments.

To investigate the effect of the *phoU2* deletion on biofilm matrix production, the release of accumulation-associated protein (Aap), polysaccharide intercellular adhesion (PIA), and extracellular DNA (eDNA) was determined for SE1457 and the Δ*phoU1* and Δ*phoU2* mutants. The production of Aap was reduced in the biofilm of the Δ*phoU2* mutant compared with that in the biofilm of the parent strain (Fig. 8A), as determined by Western blotting with monoclonal antibody 18B6 (MAb_18B6_) against the Aap protein B repeat region. PIA production was similar in the Δ*phoU2* mutant biofilm and the parent strain, as determined semiquantitatively with a wheat germ agglutinin (WGA)-horseradish peroxidase (HRP) conjugate using a dot blot 96 system (Fig. 8B). The relative concentration of eDNA in the 24-h-old biofilm of the Δ*phoU2* mutant was similar to that in the parent strain (Fig. 8C). All *P* values were >0.05, so there were no differences in the levels of transcription of *serp0306*, *leuA*, or *lysA* between biofilm bacteria of the PhoU mutants and the parent strain. The levels of PIA, Aap, and eDNA production were similar in the Δ*phoU1* mutant and the parent strain.

### Antibiotic tolerance of the Δ*phoU1* and Δ*phoU2* mutants.

Antibiotic-tolerant bacteria were identified using a modification of a procedure described by Li and Zhang (5). Both the MIC and the minimal bactericidal concentration (MBC) for the Δ*phoU1* and Δ*phoU2* mutants were similar to those for the parent strain. To determine the antibiotic concentrations that ensured that only drug-tolerant bacterial cells survived, killing curves were determined for each antibiotic used in the present study. To detect the drug tolerance of the bacterial mutants, in which the size of the population of bacteria was not decreased with an increase in the antibiotic concentration, the concentrations of antibiotics were as follows: vancomycin, 75 μg/ml (96× MIC); levofloxacin, 75 μg/ml (128× MIC); amikacin, 50 μg/ml (128× MIC).

An overnight culture (16 h) of S. epidermidis was inoculated into fresh TSB (at 1:100) containing the antibiotics at the specific concentrations, and the culture was incubated at 37°C for 5 days. At different time points, the surviving bacteria were counted. After exposure to the three antibiotics for 72 h, the antibiotic tolerance of the Δ*phoU2* mutant was dramatically reduced, and no viable bacteria were detected. In contrast, the Δ*phoU1* mutant displayed an antibiotic tolerance similar to that of the parent strain (greater than 10^4^ CFU) (Table 1). The complementation of the Δ*phoU2* mutant strain restored the antibiotic tolerance.

**TABLE 1:** Survival of the Δ*phoU1* and Δ*phoU2* mutants and the parent strain with antibiotic exposure over time^*a*^

Time point	No. of CFU/ml														
	SE1457			Δ*phoU1* mutant			Δ*phoU2* mutant			Δ*phoU2*/pCN51::*phoU2* strain			Δ*phoU2*/pCN51 strain		
	Van	Lev	Ami	Van	Lev	Ami	Van	Lev	Ami	Van	Lev	Ami	Van	Lev	Ami
Start	8 × 10^9^	8 × 10^9^	8 × 10^9^	8.7 × 10^9^	8.7 × 10^9^	8.7 × 10^9^	9 × 10^9^	9 × 10^9^	9 × 10^9^	8.5 × 10^9^	8.5 × 10^9^	8.5 × 10^9^	8.7 × 10^9^	8.7 × 10^9^	8.7 × 10^9^
12 h	6 × 10^6^	4 × 10^6^	7.2 × 10^6^	8 × 10^6^	7.8 × 10^6^	4.4 × 10^6^	8 × 10^5^	5 × 10^4^	6 × 10^5^	6.8 × 10^6^	5 × 10^6^	7.8 × 10^6^	7.6 × 10^6^	5.7 × 10^4^	4 × 10^5^
24 h	3.2 × 10^6^	3.6 × 10^6^	5 × 10^5^	6 × 10^6^	5 × 10^6^	4 × 10^6^	5 × 10^5^	2 × 10^3^	1 × 10^5^	4 × 10^6^	3.5 × 10^6^	2 × 10^6^	3.5 × 10^5^	5 × 10^3^	2.4 × 10^4^
36 h	9.2 × 10^5^	7 × 10^5^	4 × 10^5^	1.2 × 10^6^	2.4 × 10^6^	2 × 10^6^	5.4 × 10^5^	0	6 × 10^4^	2.2 × 10^6^	8 × 10^5^	7 × 10^5^	4 × 10^4^	0	2 × 10^3^
48 h	1.3 × 10^6^	5 × 10^5^	8.8 × 10^4^	1.28 × 10^6^	3 × 10^6^	5 × 10^5^	4 × 10^4^	0	1.2 × 10^4^	7 × 10^5^	8 × 10^5^	4.5 × 10^5^	2.4 × 10^3^	0	0
72 h	4.4 × 10^6^	2 × 10^5^	6 × 10^4^	2.57 × 10^6^	5 × 10^5^	4 × 10^5^	0	0	0	5 × 10^5^	4.6 × 10^5^	9 × 10^4^	0	0	0
96 h	6 × 10^6^	7 × 10^5^	4 × 10^4^	9 × 10^6^	6.6 × 10^5^	1 × 10^5^	0	0	0	7.2 × 10^5^	5 × 10^5^	7.5 × 10^4^	0	0	0
120 h	1.9 × 10^5^	6 × 10^5^	1.9 × 10^4^	6 × 10^6^	4 × 10^5^	8 × 10^4^	0	0	0	2.4 × 10^5^	4.5 × 10^5^	5 × 10^4^	0	0	0

a The susceptibilities of stationary-phase cultures of the Δ*phoU1* and Δ*phoU2* mutants and the SE1457 parent strain to vancomycin (Van; 75 μg/ml), levofloxacin (Lev; 75 μg/ml), and amikacin (Ami; 50 μg/ml) were determined. The numbers of CFU were determined at different times of exposure of stationary-phase cultures of these strains to the indicated antibiotics.

### Comparison of the transcriptomes of the Δ*phoU2* mutant and the parent strain.

To compare the transcriptional profile of the Δ*phoU1* or Δ*phoU2* mutant with that of SE1457, RNA was extracted from logarithmic-phase (6 h) and stationary-phase (10 h) bacteria and detected by transcriptome sequencing (RNA-Seq). The sequencing libraries were prepared in triplicate for the Δ*phoU1* and Δ*phoU2* mutants and the parent strain. For each biological replicate, 10 million raw reads were generated. After the removal of ambiguous and low-quality reads, more than 90% of the reads mapped to strain ATCC 35984.

A gene with a false discovery rate (FDR)-adjusted *P* value of less than 0.05 (*t* test), a *q* value of less than 0.05, and at least a 1.5-fold change in the transcript level between the mutant and the parent strain was considered to be differentially expressed. In logarithmic phase (6 h), 945 genes were identified to be differentially expressed between the Δ*phoU2* mutant and the parent strain; among these, 474 genes were upregulated and 471 were downregulated. In the stationary phase (10 h), 995 DEGs were identified; among these, 716 genes were upregulated and 279 were downregulated. By comparison and analysis, 439 DEGs were identified in the different phases of both the Δ*phoU2* mutant and the parent strain. Among these genes, 256 showed the same expression tendency. However, there were only 92 genes differentially expressed between the Δ*phoU1* mutant and the parent strain during logarithmic phase and 2 DEGs during the stationary phase. Therefore, we focused on analyzing the genes differentially expressed between the Δ*phoU2* mutant and the parent strain. We selected 70 of the DEGs for validation by RT-qPCR. Among them, the transcription of 62 genes in the Δ*phoU2* mutant was consistent with the tendency observed by RNA-Seq. The other 8 genes determined by RT-qPCR displayed a fold change in expression below the cutoff value of 2.

Among the DEGs, the transcription of *yycFG*, an essential two-component system for regulating bacterial growth, in the Δ*phoU2* mutant was downregulated 3-fold compared with its expression in the parent strain (15). Transcription of the anaerobic growth-regulated genes *pflA*, *nrdD*, and *nrdG* was downregulated in the Δ*phoU2* mutant (16–19). The expression of *rsbU* (a sigma factor B regulatory protein), which is involved in biofilm formation, was also downregulated (20). Transcription of the autolysis genes *ssaA* (*serp2136*, *serp1880*, *serp1884*, *serp2120*) and *serp0318* was upregulated in the Δ*phoU2* mutant, while the gene for autolysin E (*atlE*) was not found among the DEGs (21–23). Expression of the *pst* operon and *phoR*, involved in inorganic phosphate metabolism, was upregulated in the Δ*phoU2* mutant, while that of the *phn* ABC transporter, which participates in phosphonate metabolism, was downregulated. The expression of ATP synthase was upregulated (Table 2).

**TABLE 2:** Genes differentially expressed between the Δ*phoU2* mutant and the parent strain

Gene function and gene	GenBank accession no. (location)	Description of product	Fold change in expression by:	
			RNA-Seq	RT-qPCR^*a*^
Growth				
*yycF*	NC_002976.3 (2591084–2591786)	DNA-binding response regulator YycF	0.35	0.25 ± 0.08
*yycG*	NC_002976.3 (2587909–2591072)	Sensor	0.33	0.31 ± 0.14
*pflA*	NC_002976.3 (2411377–2412133)	Pyruvate formate-lyase-activating enzyme	0.14	0.12 ± 0.02
*nrdG*	NC_002976.3 (2215127–2217511)	Anaerobic ribonucleoside triphosphate reductase-activating protein	0.31	ND
*nrdD*	NC_002976.3 (2215127–2217511)	Anaerobic ribonucleoside triphosphate reductase	0.27	0.30 ± 0.14
Biofilm formation				
*icaR*	NC_002976.3 (2333497–2334055)	Intercellular adhesion regulator	4.58	4.20 ± 1.14
*serp0719*	NC_002976.3 (713668–716143)	Cell wall surface anchor family protein	0.18	0.13 ± 0.04
*rsbU*	NC_002976.3 (1724456–1725458)	Sigma factor B regulatory protein	0.31	ND
Autolysis				
*serp1880*	NC_002976.3 (1904430–1905204)	Secretory antigen precursor SsaA	3.56	ND
*serp1884*	NC_002976.3 (1909382–1909856)	Secretory antigen precursor SsaA	9.47	ND
*serp2120*	NC_002976.3 (2144621–2145053)	Secretory antigen precursor SsaA-related protein	1.92	ND
*serp2136*	NC_002976.3 (2161088–2161862)	Secretory antigen precursor SsaA	3.03	ND
*serp0318*	NC_002976.3 (322333–323134)	LysM domain-containing protein	1.86	ND
Phosphate transport system				
*serp0956*	NC_002976.3 (972985–973633)	Phosphate transport system regulatory protein PhoU	5.10	48.95 ± 6.43
*serp0957*	NC_002976.3 (973639–974515)	Phosphate transporter ATP-binding protein	3.75	67.96 ± 12.14
*serp0958*	NC_002976.3 (974602–975508)	Phosphate ABC transporter permease	2.29	ND
*serp0959*	NC_002976.3 (975509–976436)	Phosphate ABC transporter permease	3.32	ND
*serp0960*	NC_002976.3 (976642–977620)	Phosphate ABC transporter phosphate-binding protein	16.46	ND
*serp2283*	NC_002976.3 (2323913–2325526)	Phosphonate ABC transporter permease	0.31	ND
*serp2284*	NC_002976.3 (2323913–2325526)	Phosphonate ABC transporter permease	0.31	ND
*serp2285*	NC_002976.3 (2325527–2326301)	Phosphonate ABC transporter ATP-binding protein	0.26	ND
*serp2286*	NC_002976.3 (2326414–2327371)	Phosphonate ABC transporter substrate-binding protein	0.27	ND
*serp0317*	NC_002976.3 (321130–322141)	Phosphate transporter family protein	0.20	0.35 ± 0.09
*malA*	NC_002976.3 (1114440–1116096)	Alpha-glucosidase	0.38	ND
*lacD*	NC_002976.3 (1838482–1839460)	Tagatose-1,6-diphosphate aldolase	0.09	ND
*lacA*	NC_002976.3 (1840938–1841367)	Galactose-6-phosphate isomerase subunit LacA	0.06	0.13 ± 0.03
*lacG*	NC_002976.3 (1834966–1836379)	6-Phospho-beta-galactosidase	0.24	0.33 ± 0.07
*galU*	NC_002976.3 (2080817–2081684)	UTP-glucose-1-phosphate uridylyltransferase	0.47	ND
*lacF*	NC_002976.3 (1838148–1838463)	PTS system, lactose-specific IIA component	0.12	ND
*serp2055*	NC_002976.3 (2078963–2080604)	Phosphoglucomutase/phosphomannomutase	0.51	ND
*lacC*	NC_002976.3 (1839463–1840396)	Tagatose-6-phosphate kinase	0.09	ND
Glycolysis/gluconeogenesis				
*gntK*	NC_002976.3 (2083340–2084882)	Gluconokinase	0.19	0.33 ± 0.12
*pgi*	NC_002976.3 (535899–537231)	Glucose-6-phosphate isomerase	0.51	ND
*fruK*	NC_002976.3 (355255–356931)	1-Phosphofructokinase	21.66	ND
*fbaA*	NC_002976.3 (1771076–1771937)	Fructose-bisphosphate aldolase	1.74	ND
*gapA2*	NC_002976.3 (1287162–1288188)	Glyceraldehyde-3-phosphate dehydrogenase	0.62	ND
*pgk*	NC_002976.3 (447676–448867)	Phosphoglycerate kinase	0.35	ND
*gpmA*	NC_002976.3 (2023859–2024546)	Phosphoglyceromutase	0.23	ND
*ppdK*	NC_002976.3 (2199426–2202054)	Pyruvate phosphate dikinase	0.10	0.03 ± 0.01
*serp2133*	NC_002976.3 (2158590–2159589)	d-Lactate dehydrogenase	1.54	ND
*serp2112*	NC_002976.3 (2135451–2136504)	Alcohol dehydrogenase	0.03	0.38 ± 0.09
*serp2076*	NC_002976.3 (2100972–2102937)	Fructose-1,6-bisphosphatase	0.39	ND
Pentose phosphate pathway				
*pgi*	NC_002976.3 (535899–537231)	Glucose-6-phosphate isomerase	0.51	ND
*xylB*	NC_002976.3 (2123183–2124674)	d-Xylulose kinase	0.63	ND
*gdh*	NC_002976.3 (1867994–1868786)	Glucose-1-dehydrogenase	3.52	ND
*deoC*	NC_002976.3 (1784622–1785397)	Deoxyribose-phosphate aldolase	0.62	ND
*prsA*	NC_002976.3 (128258–129224)	Ribose-phosphate pyrophosphokinase	0.62	ND
*tkt*	NC_002976.3 (920891–922880)	Transketolase	0.59	ND
*serp2076*	NC_002976.3 (2100972–2102937)	Fructose-1,6-bisphosphatase	0.39	ND
*gntK*	NC_002976.3 (2083340–2084882)	Gluconokinase	0.19	ND
*deoB*	NC_002976.3 (1781738–1783276)	Phosphopentomutase	0.62	ND
*zwf-2*	NC_002976.3 (1111611–1113096)	Glucose-6-phosphate 1-dehydrogenase	0.56	ND
*serp2055*	NC_002976.3 (2078963–2080604)	Phosphoglucomutase/phosphomannomutase	0.51	ND
*rpe*	NC_002976.3 (788507–789152)	Ribulose-phosphate 3-epimerase	0.65	ND
*acnA*	NC_002976.3 (932350–935056)	Aconitate hydratase	0.64	ND
*icd*	NC_002976.3 (1296194–1297463)	Isocitrate dehydrogenase	0.46	ND
*serp2324*	NC_002976.3 (2360268–2361546)	Branched-chain alpha-keto acid dehydrogenase subunit E2	0.24	ND
*sucC*	NC_002976.3 (815833–817000)	Succinyl coenzyme A synthetase subunit beta	1.78	ND
*sdhB*	NC_002976.3 (730805–733414)	Succinate dehydrogenase iron-sulfur subunit	0.20	ND
*sdhA*	NC_002976.3 (2118832–2119732)	Succinate dehydrogenase flavoprotein subunit	0.17	ND
*fumC*	NC_002976.3 (1444325–1445711)	Fumarate hydratase	0.51	ND
*sucA*	NC_002976.3 (1002484–1005289)	2-Oxoglutarate dehydrogenase E1	0.44	ND
*sucB*	NC_002976.3 (1001203–1002466)	Dihydrolipoamide succinyltransferase	0.25	ND
*mqo-3*	NC_002976.3 (2350000–2351497)	Malate:quinone oxidoreductase	0.43	ND
*mqo-1*	NC_002976.3 (1970555–1972034)	Malate:quinone oxidoreductase	0.48	ND
*pckA*	NC_002976.3 (1409888–1411481)	Phosphoenolpyruvate carboxykinase	0.53	ND
*serp0857*	NC_002976.3 (868756–869623)	2-Oxoglutarate ferredoxin oxidoreductase subunit beta	0.56	ND
*serp0856*	NC_002976.3 (866995–868756)	Pyruvate ferredoxin oxidoreductase, alpha subunit	0.44	ND
*serp2325*	NC_002976.3 (2361559–2362600)	Acetoin dehydrogenase, E1 component, beta subunit	0.33	ND
*serp2327*	NC_002976.3 (2363665–2365018)	Acetoin dehydrogenase, E3 component, dihydrolipoamide dehydrogenase	1.55	ND
*serp1076*	NC_002976.3 (1120885–1122205)	2-Oxoisovalerate dehydrogenase E2	0.51	ND
*serp1077*	NC_002976.3 (1122217–1124192)	2-Oxoisovalerate dehydrogenase E1	0.40	ND
*serp1078*	NC_002976.3 (1122217–1124192)	2-Oxoisovalerate dehydrogenase E1	0.32	ND
*lpdA*	NC_002976.3 (1124206–1125628)	2-Oxoisovalerate dehydrogenase E3	0.34	ND
*serp2381*	NC_002976.3 (2428108–2431126)	NADH:flavin oxidoreductase/fumarate reductase, flavoprotein subunit	0.02	0.082

a qRT-PCR data are given as the means ± standard deviations of the results from three independent experiment. ND, not done.

Using KEGG analysis, the genes differentially expressed between the Δ*phoU2* mutant and the parent strain were involved not only in phosphate metabolism, bacterial growth, and biofilm formation but also in various pathways or processes, such as the pentose phosphate pathway, galactose metabolism, the TCA cycle, glycolysis and gluconeogenesis, respiratory chain reactions, ABC transporter activity, the phosphotransferase (PTS) system, the urea cycle, and ribosome protein production (Table 2).

**FIG 9:**
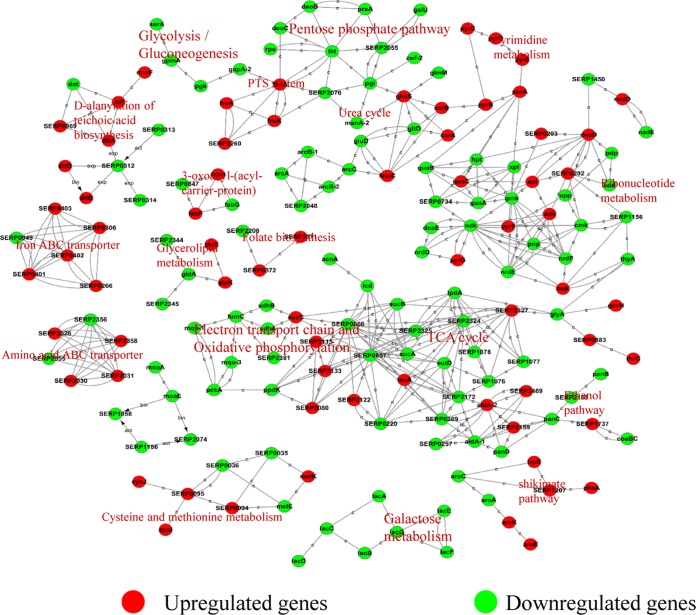
Interaction networks between DEGs identified by transcriptome comparison. The proteins encoded by DEGs (red, green), which were identified by comparison (at 6 h) of the transcriptomes of the Δ*phoU2* mutant and the parent strain, were extracted to construct a protein-protein interaction network. The lines in the network represent protein-protein interactions, including binding/association, phosphorylation, activation, and inhibition. Proteins encoded by upregulated and downregulated DEGs are indicated in red and green, respectively.

To further determine the links between the DEGs and the biological behavior in the Δ*phoU2* mutant, a protein-protein interaction (PPI) network of DEGs based on the KEGG database (http://www.genome.jp/kegg/pathway.html) was constructed using Cytoscape software. The proteins encoded by DEGs were pooled with 174 proteins involved in various metabolic pathways. The expression of genes for proteins involved in the pentose phosphate pathway, galactose metabolism, and amino acid synthesis and TCA cycle genes was downregulated (Fig. 9). In the pentose phosphate pathway, NADP (NADPH) and pentose are important products. NADPH plays a crucial role in coping with oxidative stress (24–29). As pentose is utilized to synthesize DNA and RNA, the absence of DNA and RNA in the Δ*phoU2* mutant leads to bacterial growth retardation.

### Biological validation of genes differentially expressed between the Δ*phoU2* mutant and the parent strain.

DEGs related to inorganic phosphate metabolism, ATP synthesis, and the pentose phosphate pathway were validated by the detection of intracellular polyP, ATP, and NADPH, respectively.

**FIG 10:**
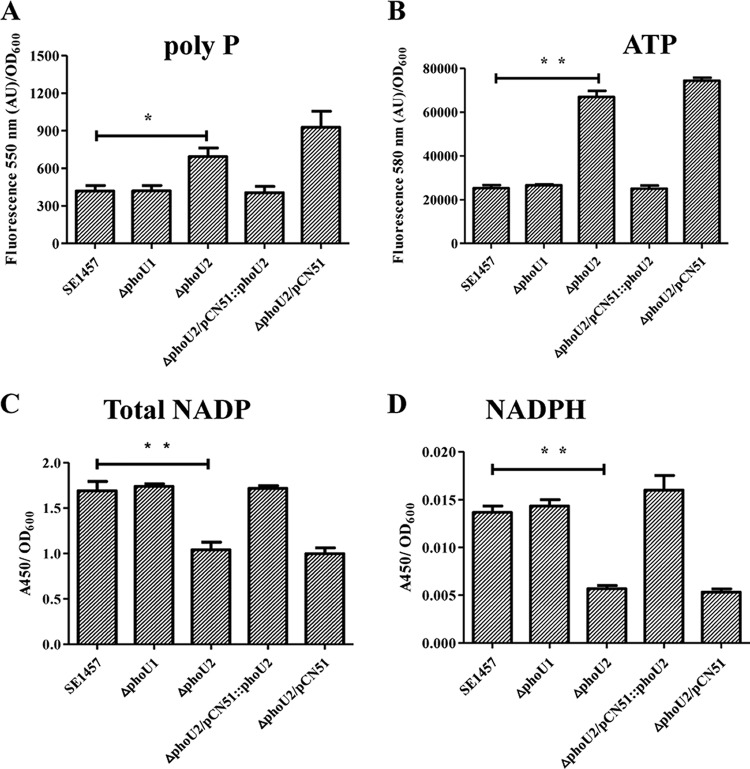
Intracellular polyP, ATP, NADP, and NADPH in the Δ*phoU2* mutant. Bacteria were grown to exponential phase in TSB medium. PolyP was assessed by measuring the fluorescence emission of the DAPI-polyP complex at 550 nm. (A) The fluorescence (in arbitrary units [AU]) of the DAPI-polyP complex was measured at 550 nm to evaluate the amount of intracellular polyP. (B) The amount of ATP was determined by measuring the fluorescence emission of the ATP complex at 587 nm. Bacteria were grown to exponential phase in TSB medium (OD_600_ = 0.5). (C and D) The amounts of total NADP and NADPH were measured using an NADP/NADPH quantification kit (Sigma). The amounts of total NADP and NADPH were determined by measuring the absorbance of the NADPH complex at 450 nm. Data (means ± SDs) are from three independent experiments. **, *P* < 0.01; *, *P* < 0.05.

The expression of DEGs involved in inorganic phosphate metabolism, the *pst* operon, and *serp0317* was upregulated in the Δ*phoU2* mutant. Inorganic phosphate (P_i_) taken up from the environment is used in bacterial metabolism, and the redundant inorganic phosphate is stored as a high-molecular-weight inorganic polyphosphate (polyP) in bacterial cells. To investigate the effects of the Δ*phoU1* and Δ*phoU2* deletions on P_i_ removal, we evaluated the levels of intracellular polyP in bacterial cells using a DAPI (4′,6-diamidino-2-phenylindole)-based fluorescence approach. The results showed that Δ*phoU2* mutant cells accumulated higher levels of polyP (1.66-fold) than the parent strain (*P* < 0.05), while the amount of intracellular polyP in the Δ*phoU1* mutant was similar to that in the parent strain (Fig. 10A). The polyP content in the *phoU2*-complemented Δ*phoU2*/pCN51::*phoU2* strain was restored to that in the parent strain, whereas the transformation of plasmid pCN51 had no effect on polyP accumulation in the Δ*phoU2* mutant.

Since polyP in cells can be converted to ATP by phosphotransferases (30) and the transcription of ATP synthase was upregulated in the Δ*phoU2* mutant, we determined the intracellular ATP levels in the Δ*phoU2* mutant, the Δ*phoU1* mutant, and the parent strain using an ATP colorimetric/fluorometric assay kit (Sigma). The intracellular ATP level in the Δ*phoU2* mutant was 2.7-fold higher than that in the parent strain (*P* < 0.01) and the Δ*phoU1* mutant (Fig. 10B). The amount of ATP recovered from the complemented Δ*phoU2*/pCN51::*phoU2* strain was similar to that recovered from the parent strain.

**FIG 11:**
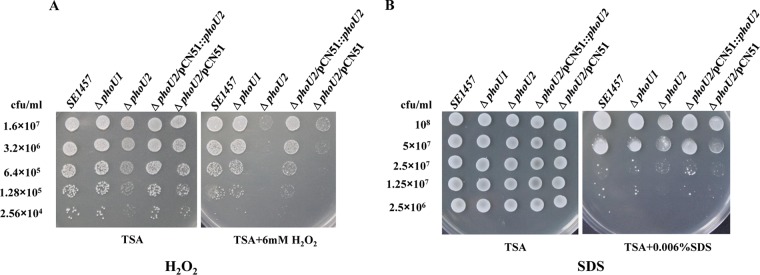
Sensitivity of the Δ*phoU1* and Δ*phoU2* mutants to H_2_O_2_ and SDS. Overnight cultures of the bacterial strains were diluted 1:200 in fresh TSB medium and incubated at 37°C with aeration for 3 h until the OD_600_ was approximately 2. After 2-fold serial dilution, each 5-μl aliquot was spotted onto a TSB agar plate containing 6 mM H_2_O_2_ or 0.006% SDS and incubated overnight at 37°C. The growth of colonies on plates containing H_2_O_2_ or SDS was photographed.

The expression of genes involved in the pentose phosphate pathway was downregulated in the Δ*phoU2* mutant. Because most of the NADP was generated via PPP and intracellular NADPH, as a reduced form of NADP, plays a very important role in protecting against the toxicity of reactive oxygen species (ROS), intracellular NADPH levels in the Δ*phoU2* mutant, the Δ*phoU1* mutant, and the parent strain were determined using an NADP/NADPH quantification kit (Sigma). The total amount of NADP in the Δ*phoU2* mutant (*A*_450_, 1.041 ± 0.085) was significantly reduced compared with that in the parent strain (*A*_450_, 1.691 ± 0.103) (*P* < 0.05) (Fig. 10C), while the amount of NADPH in the Δ*phoU2* mutant (*A*_450_, 0.014 ± 0.001) was 3-fold lower than that in the parent strain (*A*_450_, 0.006 ± 0.001) (*P* < 0.01) (Fig. 10D). The amount of total NADP or NADPH in the *phoU2*-complemented Δ*phoU2*/pCN51::*phoU2* strain was restored to the level in the parent strain. The reduced content of NADPH in the Δ*phoU2* mutant compared with that in the parent strain may have led to the weakened capacity of the Δ*phoU2* mutant to withstand oxidative stress. Then, the effects of *phoU2* on the bacterial response to H_2_O_2_ were investigated. SDS was also determined to be another stress control. Different concentrations of the bacterial culture were spotted onto TSA containing 6 mM H_2_O_2_ or 0.006% SDS (8, 31). The Δ*phoU2* mutant displayed higher sensitivity to H_2_O_2_, and no bacterial survival was detected when 6.4 × 10^5^ CFU was spotted onto plates containing 6 mM H_2_O_2_ (Fig. 11A); bacteria of the parent strain were observed at 2.56 × 10^4^ CFU. Complementation of the Δ*phoU2* mutant could restore sensitivity to H_2_O_2_, the level of which reached that of the parent strain. However, the sensitivity of the Δ*phoU1* mutant to H_2_O_2_ was similar to that of the parent strain. Both the Δ*phoU1* mutant and the Δ*phoU2* mutant displayed a level of sensitivity to SDS similar to that of the parent strain (Fig. 11B).

## DISCUSSION

PhoU in E. coli has been identified to be a regulator of phosphate uptake (32–34). Increasing attention has been focused on the regulation of PhoU in bacterial drug tolerance and the stress response. On the basis of the conserved motif of the PhoU protein, two PhoU gene homologs (*serp0956* and *serp0316*) have been identified in S. epidermidis and named *phoU1* and *phoU2*, respectively. *phoU1* is located in the *pst* operon and shares a high degree of homology with *phoU* of E. coli. *phoU2* (*serp0316*) is cotranscribed with *serp0317* (see Fig. S1 in the supplemental material). *serp0317* is another inorganic phosphate transport gene with homology to *pit* in E. coli (35, 36). Using bioinformatics analysis, the genome of S. aureus (NCTC8325) was found to contain two PhoU gene homologs (*SAOUHSC_01384* and *SAOUHSC_00669*). *phoU2* of S. epidermidis shares 86% identity with *SAOUHSC_00669* at the nucleotide sequence level and PhoU2 shares 97% identity at the amino acid sequence level with SAOUHSC_00669 in S. aureus. *SAOUHSC_00669* has been investigated as a gene required for the sensitivity of an S. aureus strain with a point mutation in *pitA* (*SAOUHSC_00670*) to daptomycin (11, 12).

The number of PhoU homologs varies in different bacterial species. In E. coli, P. aeruginosa, and Streptococcus pneumoniae, there is only one PhoU, while in T. maritima, M. tuberculosis, M. marinum, S. aureus, and S. epidermidis, two PhoU homologs have been identified. In E. coli, *phoU* plays an important role in polyP accumulation (34) and the formation of multidrug-resistant bacteria (5, 34). In P. aeruginosa, PhoU is a negative regulator of intracellular ppGpp and polyP. However, *phoU* mutation has no effect on P. aeruginosa biofilm formation (8). In species of mycobacteria, there are two *phoU* homologs, *phoY1* and *phoY2. phoY2* has been investigated as the functional homolog of *phoU*, regulating the generation of multidrug-tolerant bacteria and maintaining metabolic homeostasis and adaptation to stress conditions in M. tuberculosis and M. marinum (6, 7).

Alignment of the amino acid sequences of PhoU1 and PhoU2 from S. epidermidis RP62A, PhoU from E. coli K-12, and the two PhoU homologs (PhoY1 and PhoY2) from M. tuberculosis H37Rv showed that the metal ion-binding sites are conserved in all the PhoU homologs (Fig. S2). To investigate the regulatory functions of the *phoU* homologs, the Δ*phoU1* and Δ*phoU2* mutants of the S. epidermidis 1457 strain were constructed and the transcriptomes of the deletion mutants and the parent strain were compared. We detected the transcription levels of *phoU1* (*SERP0956*) and *phoU2* (*SERP0316*) in SE1457 by RT-qPCR at different time point (4 h, 6 h, 8 h, 10 h, and 12 h), and both *phoU1* and *phoU2* of SE1457 were expressed at the time points evaluated (Fig. S1). In logarithmic phase (6 h), 945 genes were differentially expressed between the Δ*phoU2* mutant and the parent strain; among these, 474 were upregulated and 471 were downregulated. However, only 92 DEGs were detected between Δ*phoU1* and the parent strain during logarithmic phase and 2 DEGs were detected during stationary phase. On the basis of Gene Ontology analysis of the genes differentially expressed between the Δ*phoU1* mutant and the parent strain, 23 DEGs that were downregulated in the Δ*phoU1* mutant were involved in translation, ribosomal structure, and biogenesis, while 69 upregulated DEGs were involved in 17 pathways or metabolic processes. However, the differences in the levels of expression of none of these DEGs were statistically significant (*P* > 0.05). It is worth mentioning that the *pst* operon was not identified among the genes differentially expressed between the Δ*phoU1* mutant and the parent strain, and the results were validated by RT-qPCR. Hence, in this study, we focused on investigating *phoU2* functions in S. epidermidis.

**FIG 12:**
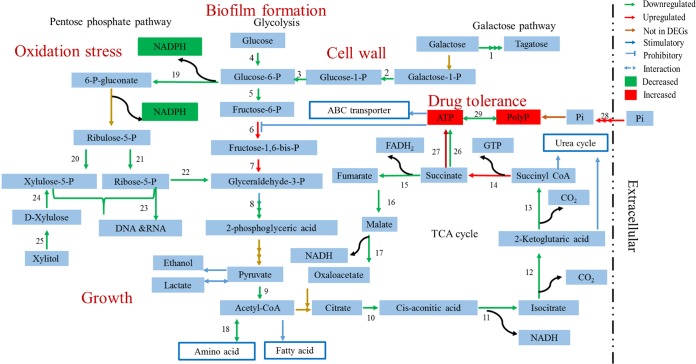
Major metabolic pathways in the Δ*phoU2* mutant revealed by DEGs. Shown are transcription of enzyme-encoding genes that were downregulated and upregulated, enzyme-encoding genes that were not found in DEGs (genes differentially expressed between the Δ*phoU2* mutant and the parent strain), stimulatory reactions, prohibitory reactions, reversible reaction interactions between the two products, and products whose amounts were increased or decreased. The numbers represent the enzyme-encoding genes listed in Table 3. P, phosphate; CoA, coenzyme A.

The Δ*phoU2* mutant of S. epidermidis displayed growth retardation under both microaerobic and oxic conditions, in accordance with the findings in an E. coli strain with a PhoU deletion (37). The deletion of *phoU1* had no effect on bacterial growth. Analysis of the DEGs (genes differentially expressed between the Δ*phoU2* mutant and the parent strain) showed that in the Δ*phoU2* mutant, the expression of *pflA* and *nrdDG* was downregulated. These genes are involved in the growth of S. epidermidis under microaerobic conditions. PflA is an enzyme activating PflB, a pyruvate formate-lyase that catalyzes the reversible conversion of pyruvate to formate, thereby producing acetyl coenzyme A. Thus, PflA plays an important role in utilization of the energy supply when pyruvate is available and favors the growth of cells under fermentation conditions (18). The protein encoded by *nrdDG* is a class III ribonucleotide reductase that catalyzes the synthesis of deoxynucleoside triphosphates (dNTPs) via the reduction of nucleoside triphosphates under anaerobic conditions (38–40). In the Δ*phoU2* mutant, the expression of *yycFG* is significantly downregulated (*P* < 0.01); *yycFG* is an essential two-component system in Gram-positive bacteria that regulates cell wall metabolism, cell division, virulence, and biofilm formation (41, 42). Expression of the essential two-component system *yycFG* was downregulated during the logarithmic phase but was not detected during the stationary phase. This finding is in accordance with the results of the assessments of the Δ*phoU2* mutant under the different growth conditions. In addition, the downregulated expression of DEGs involved in the TCA cycle, glycolysis and gluconeogenesis, and the pentose phosphate pathway may promote growth retardation (Fig. 12 and Table 3). The growth retardation of the Δ*phoU2* mutant did not persist to stationary phase, as there were no differences in the CFU counts from those of the parent strain in stationary phase.

**TABLE 3:** DEGs involved in major metabolic pathway

DEG no.	Gene name(s)	Enzyme(s) encoded
1	*lacA*, *lacD*, *lacG*	Galactose-6-phosphate isomerase subunit LacA, tagatose-1,6-diphosphate aldolase, 6-phospho-beta-galactosidase
2	*galU*	UTP-glucose-1-phosphate uridylyltransferase
3	*serp2055*	Phosphoglucomutase/phosphomannomutase
4	*gntK*	Gluconokinase
5	*pgi*	Glucose-6-phosphate isomerase
6	*fruK*	1-Phosphofructokinase
7	*fbaA*	Fructose-bisphosphate aldolase
8	*gapA2*, *pgk*, *gpmA*	Glyceraldehyde-3-phosphate dehydrogenase, phosphoglycerate kinase, phosphoglyceromutase
9	*serp0856*, *serp0857*	2-Oxoglutarate ferredoxin oxidoreductase subunit beta, pyruvate ferredoxin oxidoreductase, alpha subunit
10	*acnA*	Aconitate hydratase
11	*acnA*	Aconitate hydratase
12	*icd*	Isocitrate dehydrogenase
13	*serp2324*	Branched-chain alpha-keto acid dehydrogenase subunit E2
14	*sucC*	Succinyl coenzyme A synthetase subunit beta
15	*sdhA*, *sdhB*	Succinate dehydrogenase iron-sulfur subunit, succinate dehydrogenase flavoprotein subunit
16	*fumC*	Fumarate hydratase
17	*mqo-1*, *mqo-3*	Malate:quinone oxidoreductase, malate:quinone oxidoreductase
18	*serp1076*, *serp1077*, *serp1078*, *lpdA*	2-Oxoisovalerate dehydrogenase E2, 2-oxoisovalerate dehydrogenase E1, 2-oxoisovalerate dehydrogenase E1, 2-oxoisovalerate dehydrogenase E3
19	*zwf-2*	Glucose-6-phosphate 1-dehydrogenase
20	*rpe*	Ribulose-phosphate 3-epimerase
21	*deoB*	Phosphopentomutase
22	*deoC*	Deoxyribose-phosphate aldolase
23	*prsA*	Ribose-phosphate pyrophosphokinase
24	*xylB*	d-Xylulose kinase
25	*tkt*	Transketolase

S. epidermidis is an important nosocomial pathogen that forms biofilms on implanted medical devices. We studied the regulatory role of *phoU2* in this respect. The Δ*phoU2* mutant exhibited impaired biofilm formation under both static and hydrodynamic conditions, in contrast to the findings obtained for P. aeruginosa. A study performed in 2015 found that inactivation of *phoU* had no effect on the formation of bacterial biofilms in P. aeruginosa (8). In the Δ*phoU2* mutant, the expression of the sigma factor B regulator gene (*rsbU*), involved in biofilm formation, is downregulated (*P* < 0.01) (20). In addition, the downregulation of genes that participate in galactose metabolism plays an essential role in Bacillus subtilis biofilm formation. The intermediate product of galactose metabolism, UDP-galactose, is used in the synthesis of exopolysaccharide (EPS) of the biofilm matrix in B. subtilis (43). In accordance with these phenomena, the production of Aap was decreased in the Δ*phoU2* mutant. Defective attachment of the Δ*phoU2* mutant could be another important reason for the reduced biofilm formation. The decreased biofilm formation was not due to growth retardation, as the number of CFU of the Δ*phoU2* mutant during the stationary phase was similar to that of the parent strain, and the time required for biofilm formation was extended to 48 h.

The downregulated expression of genes involved in the TCA cycle, glycolysis, and gluconeogenesis resulted in a lack of polysaccharide, which was also responsible for the biofilm formation defect in the Δ*phoU2* mutant.

The Δ*phoU2* mutant of S. epidermidis displayed a rough cell wall and rapid autolysis. The rough cell wall may indicate that the cell wall of the Δ*phoU2* mutant was incomplete, potentially due to the low expression levels of the galactose metabolism genes. The intermediate products of galactose metabolism are involved in the synthesis of peptidoglycan. Peptidoglycan, which is also known as murein, is the most important component of Gram-positive bacterial cell walls (Fig. 12). In addition to the autolysis genes, *ssaA* and *serp0318* were upregulated in the Δ*phoU2* mutant; the incomplete cell wall may have led to the rapid autolysis in the Δ*phoU2* mutant (22, 23). Besides the peptidoglycan, the level of another component of the cell wall, teichoic acid, may also have been decreased in the Δ*phoU2* mutant. Teichoic acids are special components in the cell wall of most Gram-positive bacteria, such as species in the genera Staphylococcus, Streptococcus, and Bacillus. Teichoic acids are bacterial copolymers of glycerol phosphate or ribitol phosphate and carbohydrates linked via phosphodiester bonds and are the essential product of glycolysis or the pentose phosphate pathway. The downregulation of glycolysis or the pentose phosphate pathway in the Δ*phoU2* mutant may reduce the level of production of teichoic acids, which would result in a rough cell surface and an increase in autolysis capacity (44, 45).

Inorganic phosphate (P_i_) is an important macronutrient for all living organisms, comprising up to 3% of the bacterial dry weight, and is involved in many important pathways (8). The expression of genes in the *pst* operon and *serp0317* was upregulated in the Δ*phoU2* mutant, which is consistent with the intracellular accumulation of polyP in the Δ*phoU2* mutant. A portion of the inorganic phosphate taken up from the environment was used for bacterial metabolism, and redundant inorganic phosphate was stored as high-molecular-weight inorganic polyphosphate (polyP) in bacterial cells (46) (Fig. 12). PolyP is a linear polymer composed of several molecules of orthophosphate (P_i_) that are linked by energy-rich phosphoanhydride bonds (30). PolyP is a known stress response molecule that accumulates in microorganisms in response to nutrient deprivation, high salt concentrations, or other environmental stress conditions (30, 47). An imbalance in the amount of intracellular polyP in E. coli elicits a defective response to oxidative, osmotic, and thermal stresses (5, 34). In M. tuberculosis, a *phoY2* transposon insertion mutant accumulates large amounts of polyP and has an increased sensitivity to thermal stress, H_2_O_2_, and antibiotics (6, 7). The characteristics of phosphate metabolism in the S. epidermidis Δ*phoU2* mutant are similar to those in *phoU* deletion mutants or insertion inactivation mutants of E. coli, P. aeruginosa, M. tuberculosis, and M. marinum (5–8).

To study the regulation of phosphate metabolism by PhoU1 and PhoU2, we determined the intracellular inorganic phosphate content and the growth of the PhoU mutants (the Δ*phoU1* and Δ*phoU2* mutants), in addition to the amount of polyP, under phosphate-limiting conditions. There were no differences in the intracellular inorganic phosphate content between the PhoU mutants and the parent strain (SE1457) (Fig. S3B). The growth curves of the PhoU mutants under P_i_-limiting conditions were similar to those of the mutants cultured in TSB medium (P_i_-replete conditions) (Fig. S3A). Comparison of the transcriptomes of the PhoU mutants and the parent strain showed that the transcription of the *pst* operon was upregulated in the Δ*phoU2* mutant, which suggests that phosphate uptake was increased in the Δ*phoU2* mutant and the extra phosphate was utilized to synthesize polyP to maintain the intracellular inorganic phosphate at a level similar to that in SE1457. However, in the Δ*phoU1* mutant there was no difference in the level of *pst* operon expression, the intracellular inorganic phosphate and polyP content, or growth under P_i_-limiting conditions compared with the findings for SE1457. These results suggest that PhoU2 but not PhoU1 plays an important role in the homeostasis of intracellular Pi.

The polyP in bacterial cells could be converted to ATP by phosphotransferases. In addition, among the genes differentially expressed between the Δ*phoU2* mutant and the parent strain, the expression of genes encoding ATP synthase was upregulated. Thus, we verified that the levels of intracellular ATP were higher in the Δ*phoU2* mutant strain than the parent strain, which is consistent with the results obtained for M. marinum (7). ATP is the most important energy-providing substrate. A high level of intracellular ATP influences the expression of many genes and metabolic pathways, such as the genes for ATP-binding cassette (ABC) transporters. ABC transporters utilize the energy from ATP binding and hydrolysis to transport various substrates, including ions, amino acids, peptides, sugars, and other molecules, across cellular membranes (48). Excessive ABC transporter activity would result in an imbalance in the uptake of substances. A high level of ATP would maintain cellular metabolism at an unhealthy active level (49). Under this condition, bacteria are easily killed by bactericidal antibiotics. Therefore, S. epidermidis Δ*phoU2* showed a reduced drug tolerance compared with the wild-type strain (Fig. 12).

On the basis of the protein-protein interaction network developed for the proteins encoded by the DEGs, 174 proteins encoded by DEGs were found to be involved in various metabolic pathways, such as the pentose phosphate pathway, the TCA cycle, and galactose metabolism. Among these, the expression of all genes that participate in the pentose phosphate pathway was downregulated in the Δ*phoU2* mutant. We found that the amount of NADPH in the Δ*phoU2* mutant decreased 3-fold compared with that in the parent strain. NADPH is produced mainly via the pentose phosphate pathway, which parallels the glycolysis pathway. The pentose phosphate pathway generates NADPH and pentoses (5-carbon sugars) as well as ribose-5-phosphate. NADPH provides the reducing equivalents for biosynthetic reactions and the oxidation-reduction involved in protection against the toxicity of reactive oxygen species (ROS), allowing the regeneration of glutathione (GSH) (24–29). The decreased NADPH in the Δ*phoU2* mutant corresponded to its high sensitivity to H_2_O_2_ (Fig. 12). The major product of PPP is ribose-5-phosphate, a precursor for the synthesis of nucleotides (DNA and RNA). The reduced synthesis of ribose-5-phosphate led to a lack of DNA and RNA, and this reduced amount of ribose-5-phosphate might be responsible for the growth retardation seen in the Δ*phoU2* mutant (Fig. 12).

To confirm the role of *phoU2* on the regulation of growth and biofilm formation in SE1457, we used the antisense technology to test the effect of silencing of *phoU2* expression on the Δ*phoU1* mutant. First, we constructed a plasmid which could express *phoU2* antisense RNA (AS-*phoU2*) in the presence of 250 ng/ml anhydrotetracycline (ATc), and the plasmid was transferred to the Δ*phoU1* mutant to obtain the AS-*phoU2* Δ*phoU1* mutant. After induction with ATc, the transcription level of *phoU2* was reduced to 10% in the AS-*phoU2* Δ*phoU1* mutant compared with that in the Δ*phoU1* mutant. The AS-*phoU2* Δ*phoU1* mutant displayed a level of growth retardation that was the same as that in the Δ*phoU2* mutant (Fig. S4A), while the level of biofilm formation was less than that in the Δ*phoU1* mutant carrying the vector pMX6 (*P* < 0.01) (Fig. S4B).

In summary, *phoU2* regulates growth, biofilm formation, oxidative stress, and drug tolerance via some important pathways and processes, including the pentose phosphate pathway, glycolysis, the TCA cycle, inorganic phosphate metabolism, galactose metabolism, and ABC transporter activity in SE1457. However, no obvious phenotype of the Δ*phoU1* mutant was observed in the presence of *phoU2* in SE1457. The mechanism of *phoU2* regulation and the interaction between *phoU1* and *phoU2* in SE1457 warrant further investigation.

The effects of PhoU1 and PhoU2 on biofilm formation and antibiotic tolerance in S. epidermidis clinical strains need to be further studied by the use of gene knockout or antisense RNA technology.

## MATERIALS AND METHODS

### Bacterial strains, plasmids, growth conditions, and chemicals.

All of the bacterial strains and plasmids used in the study are shown in Table 4. The biofilm-positive strain S. epidermidis ATCC 35984 (RP62A; GenBank accession number NC_002976) and non-biofilm-forming strain ATCC 12228 (GenBank accession number NC_004461) were purchased from the American Type Culture Collection (ATCC; Manassas, VA) (50). S. epidermidis 1457 (SE1457) and S. aureus RN4220 were provided by Gao Fu from the University of Hong Kong. The S. epidermidis strains and S. aureus RN4220 were cultured in tryptic soya broth (TSB; Oxoid, Basingstoke, UK) at 37°C with shaking at 220 rpm. Glucose was added to the TSB medium at a concentration of 0.5% for the detection of biofilm formation. Electroporation was used for plasmid transformation, and B2 medium (1% casein hydrolysate, 2.5% yeast extract, 0.5% glucose, 2.5% NaCl, 0.1% K_2_HPO_4_, pH 7.5) was used for the recovery of bacteria. The antibiotics used in this study were purchased from Sigma Chemical Co. (Los Angeles, CA, USA) and used at concentrations of 10 mg/liter for chloramphenicol, 100 mg/liter for ampicillin, 50 ng/ml for anhydrotetracycline, and 10 mg/liter for erythromycin.

**TABLE 4:** Bacterial strains and plasmids used in the present study

Bacterial strain or plasmid	Description	Source
Bacterial strains		
S. epidermidis 1457	Clinical strain, biofilm positive	
Δ*phoU1* mutant	*phoU1* deletion mutant obtained using SE1457 as the parent strain	This study
Δ*phoU2* mutant	*phoU2* deletion mutant obtained using SE1457 as the parent strain	This study
Δ*phoU2*/pCN51::*phoU2*	*phoU2* mutant complemented with plasmid pCN51 harboring the *phoU2* gene	This study
Δ*phoU2*/pCN51	*phoU2* mutant complemented with plasmid pCN51	This study
S. epidermidis RP62A	Standard strain, biofilm positive	ATCC
S. epidermidis ATCC 12228	Standard strain, biofilm negative	ATCC
S. aureus RN4220	Restriction-deficient strain permitting shuttle of a plasmid modified by its host specificity determination from Gram-negative to Gram-positive bacteria	Gao Fu, University of Hong Kong
Plasmids		
pKOR1	Temp-sensitive E. coli (Amp^r^)-Staphylococcus (Cm^r^) shuttle vector	Li Min, Institute of Antibiotics, Huashan Hospital
pKOR1-Δ*phoU1*	Recombinant plasmid	This study
pKOR1-Δ*phoU2*	Recombinant plasmid	This study
pCN51	A Cd^2+^-inducible shuttle plasmid, Erm^r^	Wageningen University, Holland
pCN51- *phoU*2	A Cd^2+^-inducible shuttle plasmid, Erm^r^; the *phoU*2 gene was cloned into plasmid pCN51	This study

### Construction of Δ*phoU1* and Δ*phoU2* mutants and complemented strains.

The *phoU1* and *phoU2* deletion mutants of the SE1457 strain were constructed by allelic replacement using the temperature-sensitive plasmid pKOR1 as described by Bae and Schneewind (51). Briefly, the upstream and downstream fragments of *phoU1* and *phoU2* were amplified by PCR and separately cloned into the pKOR1 vector, resulting in pKOR1-Δ*phoU1* and pKOR1-Δ*phoU2*. The recombinant plasmid pKOR1-Δ*phoU* was successively transferred into E. coli DH5α, S. aureus RN4220, and then SE1457, followed by allelic replacement as described previously (52, 53). The *phoU1*and *phoU2* deletion mutants were verified by PCR, RT-qPCR, and direct sequencing and are referred to as the Δ*phoU1* and Δ*phoU2* mutants. The complemented Δ*phoU2* mutant strain was constructed using a shuttle vector, pCN51 (54). The *phoU2* gene and its Shine-Dalgarno region were amplified by PCR using primers *phoU2*-BamHI-F/*phoU2*-KpnI-R, whose sequences are provided in Table 5. Plasmid pCN51-*phoU2* was used for complementation and was constructed by inserting a fragment of the digested PCR products of the *phoU2* gene with BamHI and KpnI (55). Plasmid pCN51-*phoU2* was transformed by electroporation into the Δ*phoU2* mutant, forming the complemented Δ*phoU2*/pCN51::*phoU2* strain. The Δ*phoU2* strain containing the empty vector pCN51 was designated the Δ*phoU2*/pCN51 mutant. The primers used in this assay are listed in Table 5.

**TABLE 5:** Primers used in this study

Primer use and primer^*a*^	Primer sequence (5′–3′)	Location^*b*^	Size of PCR product (bp)	Note^*c*^
Construction of Δ*phoU1* mutant				
*phoU1* us-F	GGGGACAAGTTTGTACAAAAAAGCAGGCTAAATGCCTCAAGCAGAATTC	974705–974724	1,061	*attB1*
*phoU1*us-R	GGGGTACCAATTGCCATTGCATCTTATCC	973625–973645		KpnI
*phoU1*ds-F	GGGGTACCGATTAAATTATCAAATCC TATTG	972969–972991	823	KpnI
*phoU1*ds-R	GGGGACCACTTTGTACAAGAAAGCTGGGTTTGCTCAGAATAAAGGAAAAG	972127–972147		*attB2*
Construction of Δ*phoU*2 mutant				
*phoU2* us-F	GGGGACAAGTTTGTACAAAAAAGCAGGCTGTTGATCGTGGTAGACCG	319402–319419	1,100	*attB1*
*phoU2* us-R	GGGGTACCCATTAAAAATCC TCCATTTTGA	320480–320501		KpnI
*phoU2* ds-F	GGGGTACCTAAGGGAGTCTTTATTTATGTC	321114–321135	959	KpnI
*phoU2* ds-R	GGGGACCACTTTGTACAAGAAAGCTGGGTCACCCATGTTACAACCATAC	322053–322072		*attB2*
Construction of Δ*phoU2* complemented strain				
*phoU2*-BamHI-F	CGCGGATCCGTAAATCAGTTCCTCA			BamHI
*phoU2*-KpnI-R	CGGGGTACCTAAATAAAGACTCCCT			KpnI
RT-qPCR				
*gyrB* F	AGAAGAGGAAGTTAGAGAAGA	2611073–2611093	168	
*gyrB* R	GCATATCC ACTGTTATATTGAAG	2610926–2610948		
*phoU1* F	CGTCTTGGTCTTCGTGTA	973556–973573	169	
*phoU1* R	CAATAGGTTGTTGTCTCGTAAT	973405–973426		
*phoU2* F	GCTGTAGGATTACTTGTAGAC	320874–320894	200	
*phoU2* R	GCTTGACACTTATCTGCTATT	321073–321053		

a Primers were designed according to the genomic sequence of S. epidermidis RP62A (GenBank accession number NC_002976). F, forward primer; R, reverse primer.

b Location of the primer in the genomic sequence of S. epidermidis RP62A.

c The underlined sequences represent the BP reaction sites or restriction enzyme sites.

### Growth curves of the Δ*phoU1* and the Δ*phoU2* mutant strains.

The SE1457, Δ*phoU1*, Δ*phoU2*, Δ*phoU2*/pCN51::*phoU2*, and Δ*phoU2*/pCN51 strains were cultured in TSB at 37°C with shaking for 12 h. Overnight cultures of the S. epidermidis strains were diluted (1:200) in 10 ml TSB in a 100-ml flask and incubated at 37°C under aerobic or microanaerobic conditions with shaking at 220 rpm. The OD_600_ was measured using a UV spectrophotometer (Eppendorf, Hamburg, Germany) (53).

### Morphology of the Δ*phoU1* and Δ*phoU2* mutants observed by TEM.

The SE1457, Δ*phoU1*, Δ*phoU2*, Δ*phoU2*/pCN51::*phoU2*, and Δ*phoU2*/pCN51 strains were cultured in TSB at 37°C for 6 h. The log-phase bacteria were rinsed with phosphate-buffered saline (PBS), prefixed with 2.5% glutaraldehyde at 4°C for 2 h, and then fixed in 1% osmium for 3 h. The fixed samples were dehydrated in a graded ethanol series, embedded in epoxy resin, and stained with uranyl acetate and lead citrate. Ultrathin sections were cut with a Leica Ultracut microtome and observed under a transmission electron microscope (TEM; Philips Tecnai-12 Biotwin). TEM micrographs were separately obtained at magnifications of ×11,500 and ×26,500 (56, 57).

### Microtiter plate assay of biofilm formation.

The biofilm-forming ability of the strains was assessed using a semiquantitative microtiter plate assay, based on the protocol described by Christensen et al. (58). Briefly, overnight cultures of the S. epidermidis strains were diluted (1:200) into fresh TSB (containing 0.5% glucose). Aliquots of the diluted cultures were inoculated into polystyrene 96-well flat-bottomed microtiter plates (Costar; Corning, USA) and incubated at 37°C for 6 h, 12 h, 24 h, or 48 h. After the planktonic cultures were decanted, the plates were gently rinsed three times with PBS. The biofilms were fixed with 99% methyl alcohol for 15 min, stained with 2% crystal violet for 5 min, rinsed with running tap water, and air dried at room temperature. The optical densities at 570 nm (OD_570_s) of the plates were then determined using a spectrophotometer (DTX880; Beckman Coulter, Fullerton, CA) (52). The biofilm formation by each strain was assessed three times using strains ATCC 12228 and ATCC 35984 as non-biofilm-forming and biofilm-forming controls, respectively.

### CLSM of biofilms.

An overnight culture of each S. epidermidis strain (1:200 dilution) was inoculated into 2 ml TSB (with 0.5% glucose supplementation) in a cell culture dish containing a glass coverslip (World Precision Instruments, USA) (59, 60). After culturing under static conditions at 37°C for 24 h, the dish was gently washed three times with saline and then stained with LIVE/DEAD reagents (1 μM SYTO9 and 1 μM propidium iodide [PI]; Thermo Fisher Scientific, Houston, TX) for 20 min and observed using a confocal laser scanning microscope (CLSM; TCS-SP5, Leica, Germany) with a 63× oil immersion objective (numerical aperture, 1.4). Photomicrographs of the biofilms were generated using Leica LAS AF software. Three-dimensional images were created using IMARIS (version 7.0.0) software (Bitplane). The live and dead bacteria were quantified using ImageJ software. Three independent experiments were performed.

### Flow-based biofilm assays.

A BioFlux 1000 system (Fluxion Biosciences) with a Leica microscope and temperature-controlled housing was used for all imaging experiments. Automated microscopy and image processing were performed using BioFlux Montage software. BioFlux 48-well plates (catalog no. 910-0047; Fluxion Biosciences) allowing up to 24 individual treatment conditions were primed with TSB from the inlet well at a shear setting of 2 dynes/cm^2^ for 10 min. Cultures of S. epidermidis were grown to mid-log phase and normalized to an OD_600_ of 0.15. Bacteria were seeded from the outlet well into the channel and viewing window at a shear setting of 2 dynes/cm^2^ for 3 s. After 1 h of incubation at 37°C, TSB was set to flow from the inlet well at a shear setting of 0.15 dyne/cm^2^ for the duration of the experiment. Images were automatically acquired every 10 min at multiple stage positions using bright-field illumination; images were also acquired in the red channel (tetramethyl rhodamine isothiocyanate [TRITC] filter set; catalog no. 86013v2; Chroma, Bellows Falls, VT) using a 200-ms exposure time. The background-corrected average pixel intensity per image was used to quantify biofilm formation (61).

### Autolysis of the Δ*phoU1* and Δ*phoU2* mutants induced by Triton X-100.

Autolysis assays were performed as described by Brunskill and Bayles with minor modifications (62). Briefly, the S. epidermidis strains were cultured in TSB (containing 1 M NaCl) to mid-exponential phase (6 h) and harvested by centrifugation (4,000 × *g*, 4°C, 15 min). After the bacterial pellets were washed with ice-cold water, they were resuspended to an OD_600_ of 1.0 in lysis buffer (50 mM Tris-HCl buffer, pH 8.0, containing 0.05% Triton X-100) and incubated at 30°C with shaking at 200 rpm. The optical density at 600 nm was measured at 30-min intervals for 3 h (59).

### Initial adherence capacity of the Δ*phoU1* and Δ*phoU2* mutants.

Primary attachment of the Δ*phoU1* and Δ*phoU2* strains to a polystyrene surface was assessed as described previously, with modifications (52, 53, 59). Briefly, overnight cultures of the SE1457, Δ*phoU1*, Δ*phoU2*, Δ*phoU2*/pCN51::*phoU2*, and Δ*phoU2*/pCN51 strains were inoculated into TSB and cultured at 37°C to logarithmic phase (OD_600_, ≈0.6). The bacterial culture was adjusted to an OD_600_ of 0.1 with PBS and inoculated into six-well plates (2 ml/well; Nunc, Roskilde, Denmark). After incubation at 37°C for 2 h, the plates were washed gently with PBS and observed under a microscope using a 40-fold objective lens. The numbers of attached cells in each photomicrograph were counted using ImageJ software, and three microscopic fields were observed per sample.

### Assay of PIA in biofilms.

Polysaccharide intercellular adhesion (PIA) in the biofilms of the Δ*phoU1* and Δ*phoU2* mutants was semiquantified by a dot blot assay with a wheat germ agglutinin (WGA)-horseradish peroxidase (HRP) conjugate as described previously (63, 64). Briefly, overnight cultures of the S. epidermidis strains were inoculated into six-well plates (Nunc) and incubated at 37°C for 24 h. Biofilms were scraped off the bottoms of the wells, resuspended in 0.5 M EDTA, and centrifuged (13,000 × *g*, 5 min) after they were heated at 100°C for 5 min. The supernatant was treated with proteinase K (20 mg/ml) at 37°C for 3 h and inactivated at 100°C for 5 min. Serial dilutions of the PIA assay extract were transferred to a nitrocellulose membrane (Millipore, Billerica, MA) using a 96-well dot blot device (Biometra GmbH, Gottingen, Germany). The air-dried membrane was blocked with 5% (wt/vol) skim milk and subsequently incubated with the WGA (3.2 μg/ml)-HRP conjugate for 1 h (Lectinotest Laboratory, Lviv, Ukraine). HRP activity was visualized by chromogenic detection using 4-chloride-1-naphthol (Sigma) as the substrate. Quantitation of PIA was represented as the highest dilution of the supernatant in which HRP was detectable.

### Detection of Aap.

Accumulation-associated protein (Aap) expression by the Δ*phoU1* and Δ*phoU2* strains was determined by Western blotting with an Aap-specific monoclonal antibody (MAb_18B6_) that was generated in our laboratory (60). Briefly, 24-h-old biofilms of the S. epidermidis strains were collected and adjusted to an identical OD_600_ after they were washed with PBS. The bacteria were treated with lysostaphin (Sigma) and centrifuged (20,000 × *g*) at 4°C for 30 min. The supernatants were separated using SDS-PAGE (7%) and blotted onto a polyvinylidene fluoride membrane (pore size, 0.45 μm; Millipore) by electrotransfer. The membrane was incubated with MAb_18B6_ (10 ng/ml) and then with goat anti-mouse IgG conjugated to HRP (Santa Cruz, Santa Cruz, CA), followed by visualization using an enhanced chemiluminescence (ECL) Western blotting system (Thermo Fisher Scientific, Waltham, MA).

### Quantification of eDNA.

The isolation of extracellular DNA (eDNA) from the biofilms was performed as described previously (52, 53). Briefly, 24-h-old biofilms cultured in a 96-well polystyrene plate were chilled at 4°C for 1 h, and EDTA was added at a final concentration of 2.5 mM. After measurement of the OD_600_ of the unwashed biofilm (biofilm biomass), eDNA extraction solution (50 mM Tris-HCl, 10 mM ETDA, 500 mM NaCl, pH 8.0) was added to the wells. The biofilms were scraped off and centrifuged (13,000 × *g*) for 5 min at 4°C. The eDNA in the supernatant was extracted with phenol-chloroform-isoamyl alcohol (25:24:1), precipitated with 100% alcohol, and resuspended in Tris-EDTA buffer. The amount of eDNA was quantified by qPCR with SYBR Premix *Ex Taq* (TaKaRa Bio, Inc., Shiga, Japan) using the primers specific for *gyrB* (gyrase B gene), *serp0306* (ferrichrome transport ATP-binding protein A gene), *leuA* (2-isopropylmalateynthase gene), and *lysA* (diaminopimelate decarboxylase A gene). Each gene was assayed in the qPCR in triplicate in three independent experiments. Relative quantitation of the eDNA in each sample was calculated as the total amount of eDNA (in nanograms) divided by the biofilm biomass (OD_600_).

### Measurement of intracellular polyP in the Δ*phoU1* and Δ*phoU2* mutants.

A DAPI (4′,6-diamidino-2-phenylindole)-based fluorescence approach was used to evaluate the amount of intracellular polyP in the S. epidermidis cell suspensions as described previously (65). Logarithmic-phase cultures of the S. epidermidis strains grown in TSB were collected by centrifugation at 4,000 × *g* for 5 min at room temperature. The pellets were washed three times with 100 mM Tris-HCl (pH 7.4) and resuspended in the same buffer to an OD_600_ of 0.2. DAPI (Beyotime) was added to a final concentration of 10 μM. The DAPI fluorescence spectra (excitation, 415 nm; emission, 450 to 650 nm) were recorded using a Cary Eclipse fluorescence spectrophotometer (Varian) after 5 min of agitation at room temperature in the dark. The fluorescence (in arbitrary units) of the DAPI-polyP complex was measured at 550 nm for evaluation of the amount of intracellular polyP.

### Detection of intracellular ATP in the Δ*phoU1* and Δ*phoU2* mutants.

The intracellular ATP levels in the Δ*phoU1* and Δ*phoU2* mutants were detected using an ATP colorimetric/fluorometric assay kit (catalog no. MAK190) from Sigma with modification for S. epidermidis. At the time points indicated above and in the relevant figures (when the OD_600_ was 0.5), the cells were pelleted by centrifugation at 4,000 × *g* at 4°C, washed with cold sterile PBS, resuspended in 1 ml of ATP extraction buffer within 10 min of initial pelleting of the culture, and lysed with 0.1 mm glass-silica beads in a BeadBeater apparatus (BioSpec). The resulting supernatant, obtained by centrifuging the samples at 20,000 × *g* for 15 min at 4°C, was filtered through a 10-kDa-cutoff filter, as suggested by the assay manufacturer, to remove enzymes that could otherwise deplete ATP.

### Quantification of intracellular NADP^+^/NADPH in the Δ*phoU1* and Δ*phoU2* mutants.

NADP^+^ and NADPH were quantified as described by Posada et al. using an NADPH/NADP^+^ kit from Sigma, which was modified for S. epidermidis (66). Because NADPH can be unstable, the cells were extracted, filtered, and assayed as quickly as possible, essentially as described above.

### Sensitivity of the Δ*phoU1* and Δ*phoU2* mutants to H_2_O_2_ and SDS.

Overnight cultures of the S. epidermidis strains were diluted 1:200 in fresh TSB medium and incubated at 37°C for 3 h until an OD_600_ of 1 was reached. After 10-fold serial dilution, 5 μl of the aliquot was spotted onto a TSB agar plate containing 6 mM H_2_O_2_ or 0.006% SDS and incubated at 37°C overnight. The bacterial colonies on the plates were photographed and counted (5, 31).

### MIC and MBC.

The MICs of the antibiotics against the five S. epidermidis strains were determined using 2-fold serial dilutions of the antibiotics in Mueller-Hinton (MH) broth (Oxoid, Basingstoke, UK) according to CLSI guidelines (67). A log-phase culture (6 h) was adjusted to a turbidity equivalent to that of a 0.5 McFarland standard (10^8^ CFU/ml), inoculated into MH broth (1:200), and then incubated at 37°C for 16 to 20 h. The MIC endpoint was defined as the lowest concentration at which there was no visible growth in the tubes. The broth containing no drugs served as a control. The minimal bactericidal concentration (MBC) values were assessed by plating 100-μl samples from each negative culture tube (tubes with no visible bacterial growth) from the MIC assays onto blank MH broth agar plates. The MBC was the concentration at which a 99.9% reduction of the original inoculum was observed.

### Determination of antibiotic-tolerant bacteria.

Different antibiotics were added to the stationary-phase cultures (16 h) of the S. epidermidis strains (final concentrations, levofloxacin at 50 mg/liter, vancomycin at 75 mg/liter, and amikacin at 50 mg/liter), and then the cultures were incubated at 37°C for 120 h without shaking. One milliliter of the bacterial culture was then washed twice with ice-cold saline and pelleted by centrifugation for 3 min at 6,000 × *g*, and the pellet was resuspended in 1 ml cold saline, which was used to generate a series of 10-fold dilutions. Triplicate 5-μl aliquots from each dilution were spotted onto TSA plates for determination of the numbers of CFU (5). Three independent experiments were performed.

### RNA isolation and RNA sequencing.

Total RNA was isolated from strain SE1457 and the Δ*phoU1* and Δ*phoU2* mutants using an RNeasy minikit (Qiagen, Hilden, Germany) according to the manufacturer's instructions. Briefly, bacterial cultures were centrifuged at 5,000 × *g* for 5 min, and then the pellets were washed twice in 0.9% saline. The culture was homogenized 5 times using 0.1-mm zirconia-silica beads in a mini-BeadBeater (Biospec, Bartlesville, OK) at 4,800 rpm for 40 s at 1-min intervals on ice. The RNA extracted using the silica-based filter was purified with phenol-chloroform-isoamyl alcohol and precipitated with absolute ethanol.

RNA-Seq was performed according to the Illumina RNA sequencing sample preparation guide, using three biological replicates for each of the S. epidermidis strains. Samples of SE1457 and Δ*phoU1* and Δ*phoU2* mutant RNA were treated with RNase-free DNase I (TaKaRa) to prevent contamination with genomic DNA. The RNA quality was evaluated using a Bioanalyzer 2100 system (Agilent Technologies Deutschland GmbH). Prior to the sequencing analysis, rRNA was removed with a RiboZero rRNA removal kit for Gram-positive organisms. After depletion of the rRNA, fragmented RNA was used as a template for PCR with random primers. The cDNA libraries were prepared by using an mRNA-Seq sample preparation kit (Illumina). The concentration of cDNA was measured using a Qubit (version 2.0) fluorometer, the fragment size (200 to 300 bp) was verified on a Bioanalyzer 2100 system, amplification was performed using an Illumina cBot system, and sequencing was performed with an Illumina HiSeq 2500 sequencer for 51 cycles; all of these procedures were performed according to the manufacturers' protocols. Raw sequencing data were processed using the data collection software provided by Illumina.

### RNA-Seq data analysis and DEG validation by RT-qPCR.

Raw sequencing reads were preprocessed by filtering out rRNA reads, sequencing adapters, short fragment reads, and other low-quality reads. The remaining reads were mapped to the reference genome of S. epidermidis RP62A at the NCBI website with Bowtie2 software (version 2.0.5) on the basis of the local alignment algorithm. Reads that aligned to multiple locations were kept (to a maximum of 20 potential positions) to assist with the construction of gene models for genes with repetitive or low-complexity features. When the reads were aligned, 1 mismatch with the reference sequence was allowed. The alignments reported using Bowtie2 software were further processed with BEDTools software to determine the transcript expression levels and their differential expression between each two of the three samples. Expression values were presented as the number of reads per kilobase of genes per million mapped reads (RPKM). Data were visualized using the Integrated Genomics Viewer. Differential expression of all the transcripts was quantified using DEGseq software (version 2.16.1), and then the fold change values were presented. In general, we recorded no distinct difference between two transcripts when their fold change value fell between 0.666 and 1.5. Concomitantly, *P* values were determined, and significance was assessed by correcting for multiple testing using, for example, Fisher's exact test and the Wilcoxon test. Differentially expressed genes with corresponding expression values were uploaded into IPA software and analyzed using the canonical pathway. We used Fisher's exact test to select significant pathways, and the significance threshold was defined by a *P* value of 0.05. The significance of the pathway was indicated by the ratio of the number of genes from the data set that mapped to the pathway versus the total number of genes present in the canonical pathway.

For validation of DEGs identified by RNA-Seq, total RNA extracted from SE1457 and the Δ*phoU1* and Δ*phoU2* mutants was treated with the reagents from a PrimeScript RT reagent kit (TaKaRa Biotechnology, Dalian, China) for DNA digestion and reverse transcribed into cDNA. qPCRs were performed using a Mastercycler RealPlex system (Eppendorf AG, Hamburg, Germany) with SYBR green PCR reagents (SYBR Premix *Ex Taq*; TaKaRa Biotechnology, Dalian, China). The amplification conditions were 95°C for 30 s and 40 cycles of 95°C for 5 s and 60°C for 34 s, followed by a melting curve analysis. *gyrB* (DNA gyrase subunit B gene) was used as a housekeeping gene to normalize the transcript levels of the genes in the qPCR. All RT-qPCRs were performed in triplicate.

### Construction of a protein-protein interaction network.

A protein-protein interaction network was constructed using Cytoscape software. A total of 174 DEGs encoding the candidate proteins involved in bacterial metabolism were extracted. The protein-protein interaction network was constructed according to the functional relationships annotated in the KEGG database (http://www.genome.jp/kegg/pathway.html). In the network, lines were used to represent protein-protein interactions, including binding/association, phosphorylation, activation, and inhibition. Proteins encoded by upregulated and downregulated DEGs are indicated in the figures in red and green, respectively.

### Statistical analysis.

Experimental data were analyzed with SPSS software and compared using the Student *t* test or one-way analysis of variance. Differences with a *P* value of <0.05 were considered statistically significant.

### Accession number(s).

The complete RNA-Seq data set is posted in the Gene Expression Omnibus database (http://www.ncbi.nlm.nih.gov/geo/) under accession numbers GSE97400 and GSE97656 for the original data set.

## Supplementary Material

Supplemental material

## References

[1] Götz F, Peters G 2000 Colonization of medical devices by coagulase-negative staphylococci, p 55–88. *In* Waldvogel FA, Bisno AL (ed), Infections associated with indwelling medical devices, 3rd ed American Society for Microbiology, Washington, DC.

[2] Maduka-Ezeh AN, Greenwood-Quaintance KE, Karau MJ, Berbari EF, Osmon DR, Hanssen AD, Steckelberg JM, Patel R 2012 Antimicrobial susceptibility and biofilm formation of Staphylococcus epidermidis small colony variants associated with prosthetic joint infection. Diagn Microbiol Infect Dis 74:224–229. doi:10.1016/j.diagmicrobio.2012.06.029. 22901790

[3] Dunne WM 2002 Bacterial adhesion: seen any good biofilms lately? Clin Microbiol Rev 15:155–166. doi:10.1128/CMR.15.2.155-166.2002. 11932228 PMC118072

[4] Rupp ME, Fey PD, Heilmann C, Gotz F 2001 Characterization of the importance of Staphylococcus epidermidis autolysin and polysaccharide intercellular adhesin in the pathogenesis of intravascular catheter-associated infection in a rat model. J Infect Dis 183:1038–1042. doi:10.1086/319279. 11237828

[5] Li Y, Zhang Y 2007 PhoU is a persistence switch involved in persister formation and tolerance to multiple antibiotics and stresses in Escherichia coli. Antimicrob Agents Chemother 51:2092–2099. doi:10.1128/AAC.00052-07. 17420206 PMC1891003

[6] Shi W, Zhang Y 2010 PhoY2 but not PhoY1 is the PhoU homologue involved in persisters in Mycobacterium tuberculosis. J Antimicrob Chemother 65:1237–1242. doi:10.1093/jac/dkq103. 20360062 PMC2868530

[7] Wang C, Mao Y, Yu J, Zhu L, Li M, Wang D, Dong D, Liu J, Gao Q 2013 PhoY2 of mycobacteria is required for metabolic homeostasis and stress response. J Bacteriol 195:243–252. doi:10.1128/JB.01556-12. 23123909 PMC3553840

[8] de Almeida LG, Ortiz JH, Schneider RP, Spira B 2015 phoU inactivation in Pseudomonas aeruginosa enhances accumulation of ppGpp and polyphosphate. Appl Environ Microbiol 81:3006–3015. doi:10.1128/AEM.04168-14. 25710363 PMC4393453

[9] Liu J, Lou Y, Yokota H, Adams PD, Kim R, Kim SH 2005 Crystal structure of a PhoU protein homologue: a new class of metalloprotein containing multinuclear iron clusters. J Biol Chem 280:15960–15966. doi:10.1074/jbc.M414117200. 15716271

[10] Chan FY, Torriani A 1996 PstB protein of the phosphate-specific transport system of Escherichia coli is an ATPase. J Bacteriol 178:3974–3977. doi:10.1128/jb.178.13.3974-3977.1996. 8682808 PMC232664

[11] Mechler L, Herbig A, Paprotka K, Fraunholz M, Nieselt K, Bertram R 2015 A novel point mutation promotes growth phase-dependent daptomycin tolerance in Staphylococcus aureus. Antimicrob Agents Chemother 59:5366–5376. doi:10.1128/AAC.00643-15. 26100694 PMC4538524

[12] Mechler L, Bonetti EJ, Reichert S, Flotenmeyer M, Schrenzel J, Bertram R, Francois P, Gotz F 2016 Daptomycin tolerance in the Staphylococcus aureus pitA6 mutant is due to upregulation of the dlt operon. Antimicrob Agents Chemother 60:2684–2691. doi:10.1128/AAC.03022-15. 26883712 PMC4862447

[13] Lewis K 2007 Persister cells, dormancy and infectious disease. Nat Rev Microbiol 5:48–56. doi:10.1038/nrmicro1557. 17143318

[14] Otto M 2008 Staphylococcal biofilms. Curr Top Microbiol Immunol 322:207–228. 18453278 10.1007/978-3-540-75418-3_10 PMC2777538

[15] Winkler ME, Hoch JA 2008 Essentiality, bypass, and targeting of the YycFG (VicRK) two-component regulatory system in gram-positive bacteria. J Bacteriol 190:2645–2648. doi:10.1128/JB.01682-07. 18245295 PMC2293224

[16] Masalha M, Borovok I, Schreiber R, Aharonowitz Y, Cohen G 2001 Analysis of transcription of the Staphylococcus aureus aerobic class Ib and anaerobic class III ribonucleotide reductase genes in response to oxygen. J Bacteriol 183:7260–7272. doi:10.1128/JB.183.24.7260-7272.2001. 11717286 PMC95576

[17] Fuchs S, Pane-Farre J, Kohler C, Hecker M, Engelmann S 2007 Anaerobic gene expression in Staphylococcus aureus. J Bacteriol 189:4275–4289. doi:10.1128/JB.00081-07. 17384184 PMC1913399

[18] Leibig M, Liebeke M, Mader D, Lalk M, Peschel A, Gotz F 2011 Pyruvate formate lyase acts as a formate supplier for metabolic processes during anaerobiosis in Staphylococcus aureus. J Bacteriol 193:952–962. doi:10.1128/JB.01161-10. 21169491 PMC3028676

[19] Wu Y, Wu Y, Zhu T, Han H, Liu H, Xu T, Francois P, Fischer A, Bai L, Gotz F, Qu D 2015 Staphylococcus epidermidis SrrAB regulates bacterial growth and biofilm formation differently under oxic and microaerobic conditions. J Bacteriol 197:459–476. doi:10.1128/JB.02231-14. 25404696 PMC4285975

[20] Kazmierczak MJ, Wiedmann M, Boor KJ 2005 Alternative sigma factors and their roles in bacterial virulence. Microbiol Mol Biol Rev 69:527–543. doi:10.1128/MMBR.69.4.527-543.2005. 16339734 PMC1306804

[21] Heilmann C, Hussain M, Peters G, Gotz F 1997 Evidence for autolysin-mediated primary attachment of Staphylococcus epidermidis to a polystyrene surface. Mol Microbiol 24:1013–1024. doi:10.1046/j.1365-2958.1997.4101774.x. 9220008

[22] Lang S, Xu J, Stuart F, Thomas RM, Vrijbloed JW, Robinson JA 2000 Analysis of antibody A6 binding to the extracellular interferon gamma receptor alpha-chain by alanine-scanning mutagenesis and random mutagenesis with phage display. Biochemistry 39:15674–15685. doi:10.1021/bi000838z. 11123892

[23] Buist G, Steen A, Kok J, Kuipers OP 2008 LysM, a widely distributed protein motif for binding to (peptido)glycans. Mol Microbiol 68:838–847. doi:10.1111/j.1365-2958.2008.06211.x. 18430080

[24] Hensley K, Robinson KA, Gabbita SP, Salsman S, Floyd RA 2000 Reactive oxygen species, cell signaling, and cell injury. Free Radic Biol Med 28:1456–1462. doi:10.1016/S0891-5849(00)00252-5. 10927169

[25] Nordberg J, Arner ES 2001 Reactive oxygen species, antioxidants, and the mammalian thioredoxin system. Free Radic Biol Med 31:1287–1312. doi:10.1016/S0891-5849(01)00724-9. 11728801

[26] Lee SM, Koh HJ, Park DC, Song BJ, Huh TL, Park JW 2002 Cytosolic NADP(+)-dependent isocitrate dehydrogenase status modulates oxidative damage to cells. Free Radic Biol Med 32:1185–1196. doi:10.1016/S0891-5849(02)00815-8. 12031902

[27] Maeng O, Kim YC, Shin HJ, Lee JO, Huh TL, Kang KI, Kim YS, Paik SG, Lee H 2004 Cytosolic NADP(+)-dependent isocitrate dehydrogenase protects macrophages from LPS-induced nitric oxide and reactive oxygen species. Biochem Biophys Res Commun 317:558–564. doi:10.1016/j.bbrc.2004.03.075. 15063794

[28] Giro M, Carrillo N, Krapp AR 2006 Glucose-6-phosphate dehydrogenase and ferredoxin-NADP(H) reductase contribute to damage repair during the soxRS response of Escherichia coli. Microbiology 152:1119–1128. doi:10.1099/mic.0.28612-0. 16549675

[29] Marino D, Gonzalez EM, Frendo P, Puppo A, Arrese-Igor C 2007 NADPH recycling systems in oxidative stressed pea nodules: a key role for the NADP^+^-dependent isocitrate dehydrogenase. Planta 225:413–421. 16896792 10.1007/s00425-006-0354-5

[30] Kornberg A 1999 Inorganic polyphosphate: a molecule of many functions. Prog Mol Subcell Biol 23:1–18. doi:10.1007/978-3-642-58444-2_1. 10448669

[31] Liu X, Sun X, Wu Y, Xie C, Zhang W, Wang D, Chen X, Qu D, Gan J, Chen H, Jiang H, Lan L, Yang CG 2013 Oxidation-sensing regulator AbfR regulates oxidative stress responses, bacterial aggregation, and biofilm formation in Staphylococcus epidermidis. J Biol Chem 288:3739–3752. doi:10.1074/jbc.M112.426205. 23271738 PMC3567629

[32] Surin BP, Rosenberg H, Cox GB 1985 Phosphate-specific transport system of Escherichia coli: nucleotide sequence and gene-polypeptide relationships. J Bacteriol 161:189–198. 3881386 10.1128/jb.161.1.189-198.1985 PMC214855

[33] Kim SK, Makino K, Amemura M, Shinagawa H, Nakata A 1993 Molecular analysis of the phoH gene, belonging to the phosphate regulon in Escherichia coli. J Bacteriol 175:1316–1324. doi:10.1128/jb.175.5.1316-1324.1993. 8444794 PMC193217

[34] Morohoshi T, Maruo T, Shirai Y, Kato J, Ikeda T, Takiguchi N, Ohtake H, Kuroda A 2002 Accumulation of inorganic polyphosphate in phoU mutants of Escherichia coli and Synechocystis sp. strain PCC6803. Appl Environ Microbiol 68:4107–4110. doi:10.1128/AEM.68.8.4107-4110.2002. 12147514 PMC124021

[35] Beard SJ, Hashim R, Wu G, Binet MR, Hughes MN, Poole RK 2000 Evidence for the transport of zinc(II) ions via the pit inorganic phosphate transport system in Escherichia coli. FEMS Microbiol Lett 184:231–235. doi:10.1111/j.1574-6968.2000.tb09019.x. 10713426

[36] Vershinina OA, Znamenskaia LV 2002 The Pho regulons of bacteria. Mikrobiologiia 71:581–595. (In Russian.) 12449623

[37] Steed PM, Wanner BL 1993 Use of the *rep* technique for allele replacement to construct mutants with deletions of the pstSCAB-phoU operon: evidence of a new role for the PhoU protein in the phosphate regulon. J Bacteriol 175:6797–6809. doi:10.1128/jb.175.21.6797-6809.1993. 8226621 PMC206803

[38] Sun G, Sharkova E, Chesnut R, Birkey S, Duggan MF, Sorokin A, Pujic P, Ehrlich SD, Hulett FM 1996 Regulators of aerobic and anaerobic respiration in Bacillus subtilis. J Bacteriol 178:1374–1385. doi:10.1128/jb.178.5.1374-1385.1996. 8631715 PMC177812

[39] Roca I, Torrents E, Sahlin M, Gibert I, Sjoberg BM 2008 NrdI essentiality for class Ib ribonucleotide reduction in Streptococcus pyogenes. J Bacteriol 190:4849–4858. doi:10.1128/JB.00185-08. 18502861 PMC2447006

[40] Kinkel TL, Roux CM, Dunman PM, Fang FC 2013 The Staphylococcus aureus SrrAB two-component system promotes resistance to nitrosative stress and hypoxia. mBio 4:e00696-13. doi:10.1128/mBio.00696-13. 24222487 PMC3892780

[41] Fabret C, Hoch JA 1998 A two-component signal transduction system essential for growth of Bacillus subtilis: implications for anti-infective therapy. J Bacteriol 180:6375–6383. 9829949 10.1128/jb.180.23.6375-6383.1998 PMC107725

[42] Dubrac S, Bisicchia P, Devine KM, Msadek T 2008 A matter of life and death: cell wall homeostasis and the WalKR (YycGF) essential signal transduction pathway. Mol Microbiol 70:1307–1322. doi:10.1111/j.1365-2958.2008.06483.x. 19019149

[43] Chai Y, Beauregard PB, Vlamakis H, Losick R, Kolter R 2012 Galactose metabolism plays a crucial role in biofilm formation by Bacillus subtilis. mBio 3:e00184-12. doi:10.1128/mBio.00184-12. 22893383 PMC3419520

[44] Bera A, Biswas R, Herbert S, Kulauzovic E, Weidenmaier C, Peschel A, Gotz F 2007 Influence of wall teichoic acid on lysozyme resistance in Staphylococcus aureus. J Bacteriol 189:280–283. doi:10.1128/JB.01221-06. 17085565 PMC1797201

[45] Schlag M, Biswas R, Krismer B, Kohler T, Zoll S, Yu W, Schwarz H, Peschel A, Gotz F 2010 Role of staphylococcal wall teichoic acid in targeting the major autolysin Atl. Mol Microbiol 75:864–873. doi:10.1111/j.1365-2958.2009.07007.x. 20105277

[46] Andrey DO, Renzoni A, Monod A, Lew DP, Cheung AL, Kelley WL 2010 Control of the Staphylococcus aureus toxic shock tst promoter by the global regulator SarA. J Bacteriol 192:6077–6085. doi:10.1128/JB.00146-10. 20870770 PMC2976458

[47] Rao NN, Gomez-Garcia MR, Kornberg A 2009 Inorganic polyphosphate: essential for growth and survival. Annu Rev Biochem 78:605–647. doi:10.1146/annurev.biochem.77.083007.093039. 19344251

[48] Davidson AL, Dassa E, Orelle C, Chen J 2008 Structure, function, and evolution of bacterial ATP-binding cassette systems. Microbiol Mol Biol Rev 72:317–364. doi:10.1128/MMBR.00031-07. 18535149 PMC2415747

[49] Conlon BP, Rowe SE, Gandt AB, Nuxoll AS, Donegan NP, Zalis EA, Clair G, Adkins JN, Cheung AL, Lewis K 2016 Persister formation in Staphylococcus aureus is associated with ATP depletion. Nat Microbiol 1:16051. doi:10.1038/nmicrobiol.2016.51. PMC4932909 27572649

[50] Gill SR, Fouts DE, Archer GL, Mongodin EF, Deboy RT, Ravel J, Paulsen IT, Kolonay JF, Brinkac L, Beanan M, Dodson RJ, Daugherty SC, Madupu R, Angiuoli SV, Durkin AS, Haft DH, Vamathevan J, Khouri H, Utterback T, Lee C, Dimitrov G, Jiang L, Qin H, Weidman J, Tran K, Kang K, Hance IR, Nelson KE, Fraser CM 2005 Insights on evolution of virulence and resistance from the complete genome analysis of an early methicillin-resistant Staphylococcus aureus strain and a biofilm-producing methicillin-resistant Staphylococcus epidermidis strain. J Bacteriol 187:2426–2438. doi:10.1128/JB.187.7.2426-2438.2005. 15774886 PMC1065214

[51] Bae T, Schneewind O 2006 Allelic replacement in Staphylococcus aureus with inducible counter-selection. Plasmid 55:58–63. doi:10.1016/j.plasmid.2005.05.005. 16051359

[52] Zhu T, Lou Q, Wu Y, Hu J, Yu F, Qu D 2010 Impact of the Staphylococcus epidermidis LytSR two-component regulatory system on murein hydrolase activity, pyruvate utilization and global transcriptional profile. BMC Microbiol 10:287. doi:10.1186/1471-2180-10-287. 21073699 PMC2996381

[53] Lou Q, Zhu T, Hu J, Ben H, Yang J, Yu F, Liu J, Wu Y, Fischer A, Francois P, Schrenzel J, Qu D 2011 Role of the SaeRS two-component regulatory system in Staphylococcus epidermidis autolysis and biofilm formation. BMC Microbiol 11:146. doi:10.1186/1471-2180-11-146. 21702925 PMC3224141

[54] Helle L, Kull M, Mayer S, Marincola G, Zelder ME, Goerke C, Wolz C, Bertram R 2011 Vectors for improved Tet repressor-dependent gradual gene induction or silencing in Staphylococcus aureus. Microbiology 157:3314–3323. doi:10.1099/mic.0.052548-0. 21921101

[55] Charpentier E, Anton AI, Barry P, Alfonso B, Fang Y, Novick RP 2004 Novel cassette-based shuttle vector system for gram-positive bacteria. Appl Environ Microbiol 70:6076–6085. doi:10.1128/AEM.70.10.6076-6085.2004. 15466553 PMC522135

[56] Gust AA, Biswas R, Lenz HD, Rauhut T, Ranf S, Kemmerling B, Gotz F, Glawischnig E, Lee J, Felix G, Nurnberger T 2007 Bacteria-derived peptidoglycans constitute pathogen-associated molecular patterns triggering innate immunity in Arabidopsis. J Biol Chem 282:32338–32348. doi:10.1074/jbc.M704886200. 17761682

[57] Vollmer W, Seligman SJ 2010 Architecture of peptidoglycan: more data and more models. Trends Microbiol 18:59–66. doi:10.1016/j.tim.2009.12.004. 20060721

[58] Christensen GD, Simpson WA, Younger JJ, Baddour LM, Barrett FF, Melton DM, Beachey EH 1985 Adherence of coagulase-negative staphylococci to plastic tissue culture plates: a quantitative model for the adherence of staphylococci to medical devices. J Clin Microbiol 22:996–1006. 3905855 10.1128/jcm.22.6.996-1006.1985 PMC271866

[59] Qin Z, Ou Y, Yang L, Zhu Y, Tolker-Nielsen T, Molin S, Qu D 2007 Role of autolysin-mediated DNA release in biofilm formation of Staphylococcus epidermidis. Microbiology 153:2083–2092. doi:10.1099/mic.0.2007/006031-0. 17600053

[60] Hu J, Xu T, Zhu T, Lou Q, Wang X, Wu Y, Huang R, Liu J, Liu H, Yu F, Ding B, Huang Y, Tong W, Qu D 2011 Monoclonal antibodies against accumulation-associated protein affect EPS biosynthesis and enhance bacterial accumulation of Staphylococcus epidermidis. PLoS One 6:e20918. doi:10.1371/journal.pone.0020918. 21687690 PMC3110253

[61] Lam H, Kesselly A, Stegalkina S, Charlebois RL, Oomen R, Kleanthous H, Yethon JA 2015 Correction for Lam et al., antibodies to PhnD inhibit staphylococcal biofilms. Infect Immun 83:2197. doi:10.1128/IAI.00285-15. 25878162 PMC4399043

[62] Brunskill EW, Bayles KW 1996 Identification and molecular characterization of a putative regulatory locus that affects autolysis in Staphylococcus aureus. J Bacteriol 178:611–618. doi:10.1128/jb.178.3.611-618.1996. 8550490 PMC177702

[63] Gerke C, Kraft A, Sussmuth R, Schweitzer O, Gotz F 1998 Characterization of the N-acetylglucosaminyltransferase activity involved in the biosynthesis of the Staphylococcus epidermidis polysaccharide intercellular adhesin. J Biol Chem 273:18586–18593. doi:10.1074/jbc.273.29.18586. 9660830

[64] Wu Y, Wang J, Xu T, Liu J, Yu W, Lou Q, Zhu T, He N, Ben H, Hu J, Gotz F, Qu D 2012 The two-component signal transduction system ArlRS regulates Staphylococcus epidermidis biofilm formation in an ica-dependent manner. PLoS One 7:e40041. doi:10.1371/journal.pone.0040041. 22848368 PMC3407220

[65] Aschar-Sobbi R, Abramov AY, Diao C, Kargacin ME, Kargacin GJ, French RJ, Pavlov E 2008 High sensitivity, quantitative measurements of polyphosphate using a new DAPI-based approach. J Fluoresc 18:859–866. doi:10.1007/s10895-008-0315-4. 18210191

[66] Posada AC, Kolar SL, Dusi RG, Francois P, Roberts AA, Hamilton CJ, Liu GY, Cheung A 2014 Importance of bacillithiol in the oxidative stress response of Staphylococcus aureus. Infect Immun 82:316–332. doi:10.1128/IAI.01074-13. 24166956 PMC3911838

[67] Turnidge J, Bordash G 2007 Statistical methods for establishing quality control ranges for antibacterial agents in Clinical and Laboratory Standards Institute susceptibility testing. Antimicrob Agents Chemother 51:2483–2488. doi:10.1128/AAC.01457-06. 17438045 PMC1913260

