# Supplementary material for: PhoU2 but Not PhoU1 as an Important Regulator of Biofilm Formation and Tolerance to Multiple Stresses by Participating in Various Fundamental Metabolic Processes in Staphylococcus epidermidis
Source: J Bacteriol. 2017 Nov 14;199(24):e00219-17. doi: 10.1128/JB.00219-17 (PMC5686610; doi:10.1128/JB.00219-17)
Supplement: Supplemental material [file supp_199_24_e00219-17__index.html]

Supplemental material 

# PhoU2 but Not PhoU1 as an Important Regulator of Biofilm Formation and Tolerance to Multiple Stresses by Participating in Various Fundamental Metabolic Processes in Staphylococcus epidermidis

## Supplemental material

- Supplemental file 1 -

  Movie S1 (Synthesis of images used to generate curves in Fig. 6)

  AVI, 4.6M
- Supplemental file 2 -

  Movie S2 (Synthesis of images used to generate curves in Fig. 6)

  AVI, 4.6M
- Supplemental file 3 -

  Movie S3 (Synthesis of images used to generate curves in Fig. 6)

  AVI, 3.4M
- Supplemental file 4 -

  Movie S4 (Synthesis of images used to generate curves in Fig. 6)

  AVI, 19M
- Supplemental file 5 -

  Movie S5 (Synthesis of images used to generate curves in Fig. 6)

  AVI, 17M
- Supplemental file 6 -

  Fig. S1 (Identification and transcription level of *phoU* operons), S2 (Conserved motif in PhoU homologs), S3 (Growth curves and intracellular Pi of ∆*phoU1* and ∆*phoU2* strains), and S4 (Growth curves and biofilm formation by AS-*phoU2* ∆*phoU1* strains on microtiter plates)

  PDF, 676K
