# Supplementary material for: PhoU2 but Not PhoU1 as an Important Regulator of Biofilm Formation and Tolerance to Multiple Stresses by Participating in Various Fundamental Metabolic Processes in Staphylococcus epidermidis
Source: J Bacteriol. 2017 Nov 14;199(24):e00219-17. doi: 10.1128/JB.00219-17 (PMC5686610; doi:10.1128/JB.00219-17)
Supplement: Supplemental material [file JB.00219-17_zjb024174594s6.pdf]

Supplemental Data

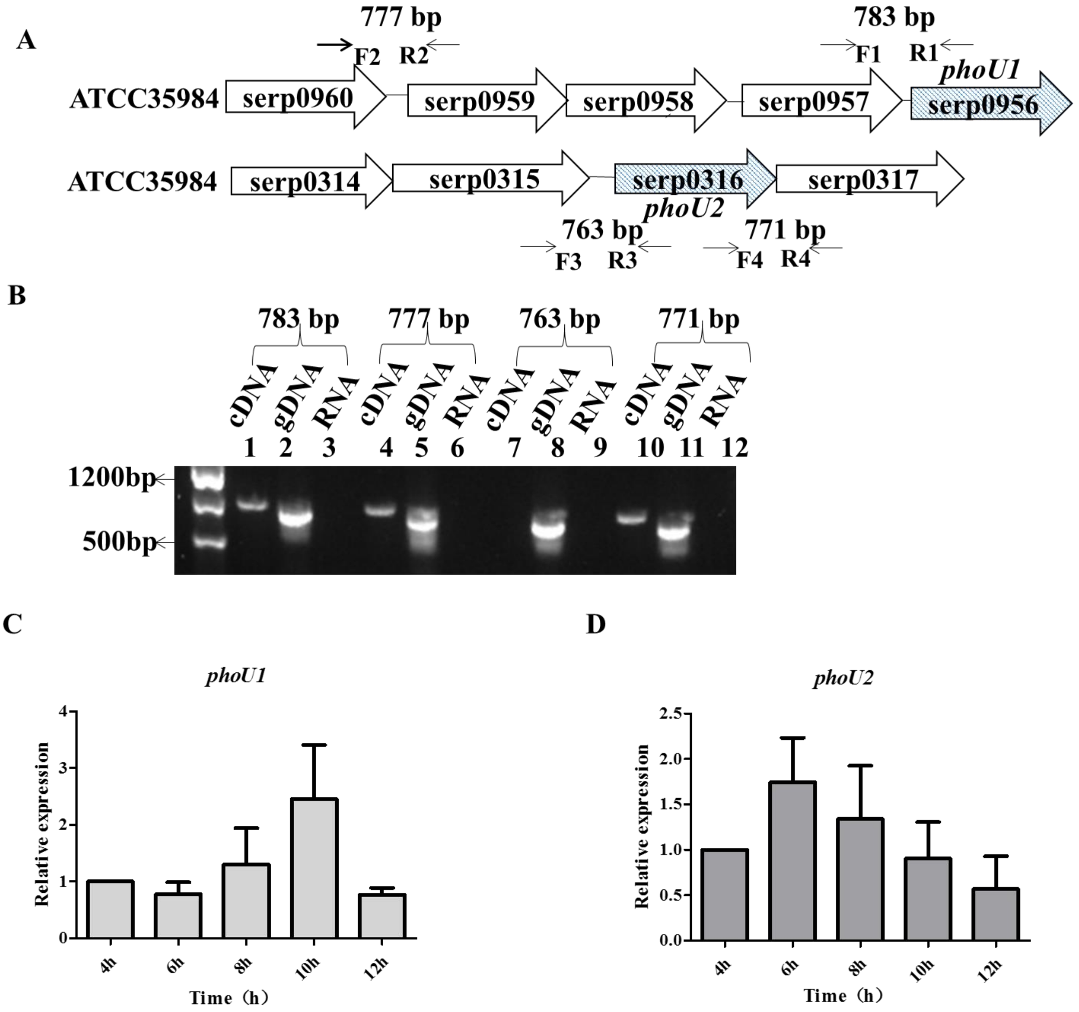

Fig. S1 Identification and transcription level of *phoU* operons. (A) Sketch map of the primers designed for the *phoU1* and *phoU2* co-transcription genes. (B) PCR product electrophoresis banding using different primers and template. In Lanes 1, 2, and 3, F1 and R1 primers were used to identify the co-transcription of *serp0956* and *serp0957*. cDNA was used as the template for PCR in Lane 1, genomic DNA in Lane 2 and RNA in Lane 3. In Lanes 4, 5, and 6, primers of F2 and R2 were used to identify the co-transcription of *serp0959* and *serp0960*. In Lanes 7, 8, and 9, the F3 and R3 primers were used to identify the co-transcription of *serp0315* and *serp0316*. In Lanes 10, 11, and 12, primers F3 and R3 were used to identify the co-transcription for *serp031*6 and

serp0317. (C) The transcription level of *phoU1* at different time point. (D) The transcription level of *phoU2* at different time point. Overnight cultures of Δ*phoU1*, Δ*phoU2* and SE1457 strains were diluted 1:200 into fresh TSB. Total RNA extracted from SE1457, Δ*phoU1* and Δ*phoU2* was treated with the PrimeScript^{TM} RT reagent kit (Takara Biotechnology, Dalian, China) for DNA digestion and reverse-transcribed into cDNA. Q-PCRs were performed using the Mastercycler realplex system (Eppendorf AG, Hamburg, Germany) with SYBR green PCR reagents (Premix EX Taq TM, Takara Biotechnology, Dalian, China).The amplification conditions were 95℃ for 30 s, 40 cycles of 95℃ for 5 s, and 60℃ for 34 s, followed by a melting curve analysis. *gyrB* (DNA gyrase subunit B) was used as a housekeeping gene to normalize the transcription levels of genes in the qPCR. All RT-qPCRs were performed in triplicate.

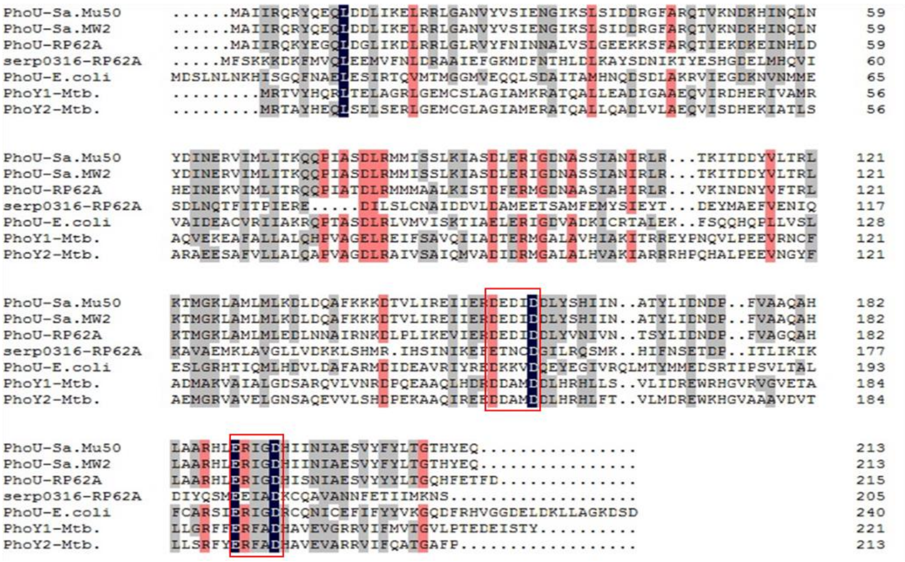

Fig S2 The conserved motif [(E(D)XXXD] in PhoU homologs. The amino acid sequence was aligned using DNAMAN software. The amino acid sequences in the red square had the conserved motif of PhoU in different bacterial species, in accordance with the reported sequences in *Thermotoga maritima*.

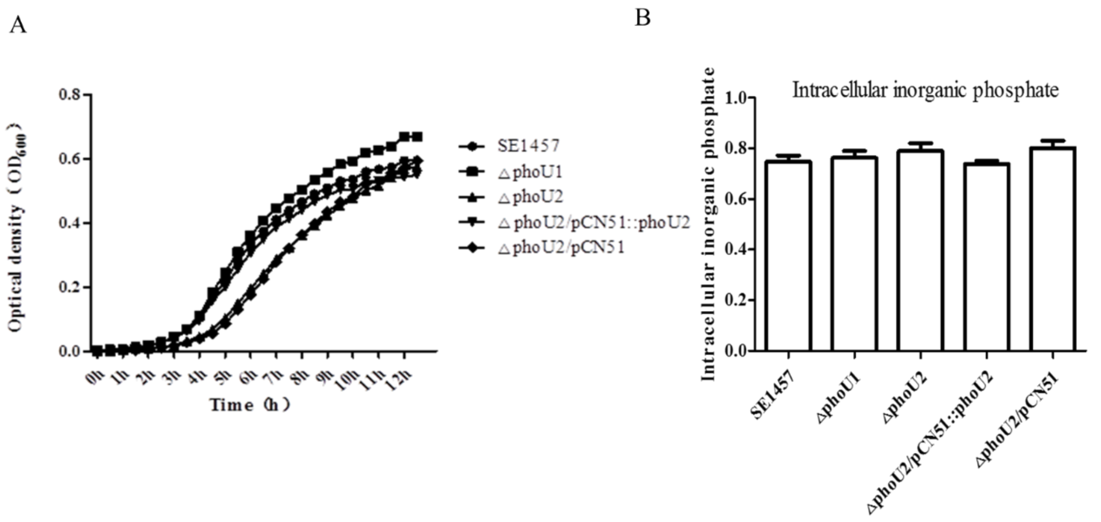

Fig. S3 (A) Growth curves of Δ*phoU1* and Δ*phoU2* under Pi-limiting conditions. Overnight cultures of Δ*phoU1*, Δ*phoU2* and SE1457 strains were diluted 1:200 into 10 ml SSM9PR (without Na_2HPO_4, KH_2PO_4) in a conical flask in a volume of 100 ml and incubated with shaking at 220 rpm. Bacterial growth was monitored by measuring the OD_{600} for 12 h. The Pi-limiting conditions was used a medium named SSM9PR which containing 1xM9 salts (without Na_2HPO_4, KH_2PO_4), 2 mM $MgSO_4$, 0.1mM $CaCl_2$, 1% glucose, 1% casaminoacids,1 mM Thiamine-HCl and 0.05mM nicotinamide. (B) Intracellular Pi of Δ*phoU1* and Δ*phoU2*. Overnight cultures of Δ*phoU1*, Δ*phoU2* and SE1457 strains were diluted 1:200 into 4 ml TSB. A commercially available kit system (no. ab65622; Abcam) was used to quantify intracellular inorganic phosphate (Pi) levels. Overnight cultures of Δ*phoU1*, Δ*phoU2* and SE1457 strains were diluted 1:200 into 4 ml TSB. After 6 h, the cultures were chilled on ice for 15 min before cells were harvested by centrifugation at 4,000 g for 10 min at 4°C. The pellet was washed twice adjusted to an OD_{600} of 1.0; 1 ml of that suspension was used for cell disruption for 30 s, in three rounds, with 0.5 ml of 0.1-mm glass zirconium/silica beads. Homogenized samples were centrifuged at 15,000 g for 15 min at 4°C. The supernatant was diluted

1:200, and Pi levels were determined according to the manufacturer's instructions. The experiments were repeated three times, and the data represent means±SD. P>0.05.

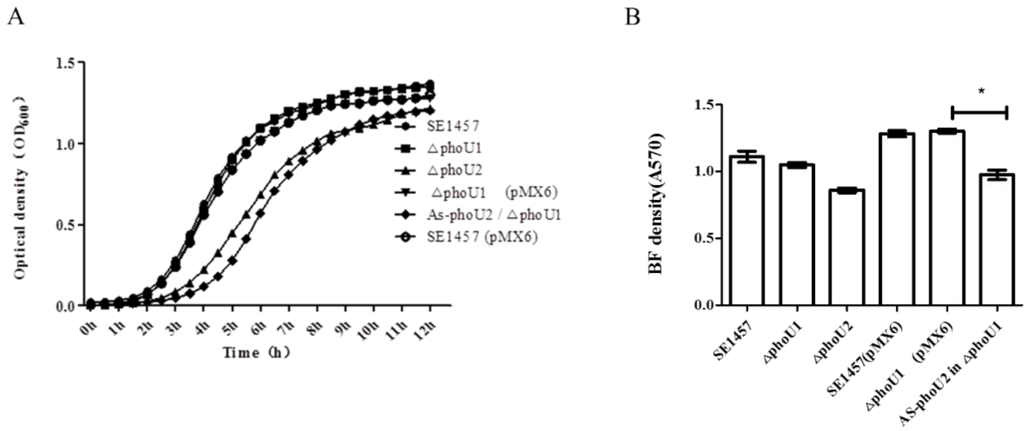

Fig. S4 (A) Growth curves of AS–*phoU2*/Δ*phoU1*. Overnight cultures of *Staphylococcus* strains were diluted 1:200 into 10 ml TSB containing 250ng/ml anhydrotetracycline and incubated with shaking at 220 rpm. Bacterial growth was monitored by measuring the OD_{600} for 12 h. (B) Biofilm formation by AS–*phoU2* / Δ*phoU1* on microtiter plates. Overnight culture of the *S. epidermidis* strains were diluted 1:200 with fresh TSB (containing 250ng/ml anhydrotetracycline), added to 96-well polystyrene plates in triplicate and cultured under static conditions for 6 h. After washing, the biofilms were stained with crystal violet. The plates were analyzed at OD_{570}. The experiments were repeated three times, and the data represent means±SD. *, $P<0.05$.
